# Characterization of *Capsicum annuum* Genetic Diversity and Population Structure Based on Parallel Polymorphism Discovery with a 30K Unigene Pepper GeneChip

**DOI:** 10.1371/journal.pone.0056200

**Published:** 2013-02-08

**Authors:** Theresa A. Hill, Hamid Ashrafi, Sebastian Reyes-Chin-Wo, JiQiang Yao, Kevin Stoffel, Maria-Jose Truco, Alexander Kozik, Richard W. Michelmore, Allen Van Deynze

**Affiliations:** 1 Seed Biotechnology Center, University of California Davis, Davis, California, United States of America; 2 Genome Center, University of California Davis, Davis, California, United States of America; 3 Department of Plant Sciences, University of California Davis, Davis, California, United States of America; University of Arizona, United States of America

## Abstract

The widely cultivated pepper, *Capsicum spp*., important as a vegetable and spice crop world-wide, is one of the most diverse crops. To enhance breeding programs, a detailed characterization of *Capsicum* diversity including morphological, geographical and molecular data is required. Currently, molecular data characterizing *Capsicum* genetic diversity is limited. The development and application of high-throughput genome-wide markers in *Capsicum* will facilitate more detailed molecular characterization of germplasm collections, genetic relationships, and the generation of ultra-high density maps. We have developed the Pepper GeneChip® array from Affymetrix for polymorphism detection and expression analysis in *Capsicum*. Probes on the array were designed from 30,815 unigenes assembled from expressed sequence tags (ESTs). Our array design provides a maximum redundancy of 13 probes per base pair position allowing integration of multiple hybridization values per position to detect single position polymorphism (SPP). Hybridization of genomic DNA from 40 diverse *C. annuum* lines, used in breeding and research programs, and a representative from three additional cultivated species (*C. frutescens, C. chinense* and *C. pubescens*) detected 33,401 SPP markers within 13,323 unigenes. Among the *C. annuum* lines, 6,426 SPPs covering 3,818 unigenes were identified. An estimated three-fold reduction in diversity was detected in non-pungent compared with pungent lines, however, we were able to detect 251 highly informative markers across these *C. annuum* lines. In addition, an 8.7 cM region without polymorphism was detected around *Pun1* in non-pungent *C. annuum*. An analysis of genetic relatedness and diversity using the software Structure revealed clustering of the germplasm which was confirmed with statistical support by principle components analysis (PCA) and phylogenetic analysis. This research demonstrates the effectiveness of parallel high-throughput discovery and application of genome-wide transcript-based markers to assess genetic and genomic features among *Capsicum annuum*.

## Introduction

Peppers, *Capsicum spp*., are grown worldwide for vegetable, spice, ornamental, medicinal and lachrymator uses and are a significant source of vitamins A and C [1]. Peppers have been found along with other food fossils from as early as 6,000 years ago and are considered the first spice to have been used by humans [2]. The genus *Capsicum* lays within the Solanoideae sub-family of the Solanaceae family and is believed to have its ancestral origins in the tropical South American region centered in what is now Bolivia [3], [4]. Currently, 38 species of *Capsicum* are reported [5]. Of these, five (*C. annuum, C. frutescens*, *C. chinense*, *C. pubescens*, and *C. baccatum*) are thought to have been domesticated through at least five independent events [6]. These domesticates are believed to be derived from three distinct genetic lineages, with *C. pubescens* and *C. baccatum* each representing independent lineages while the domesticated taxa *C. annuum*, *C. frutescens* and *C. chinense* are considered members of a species complex that were each independently derived from wild progenitors that may or may not be independent species [3], [7]. This is supported by the ability to make interspecific hybrids between these three *Capsicum* species. The most commonly cultivated species worldwide, *C. annuum*, was domesticated in Mexico from the wild bird pepper. Its predominance among cultivated species globally has been attributed to it being the first *Capsicum* introduced to Europe, by Columbus and other early new world explorers, rather than superior agronomic or consumer traits [8], [9]. *C. annuum* has subsequently become one of the most important spice commodities as well as an important vegetable crop globally [1], [2].

The wild progenitor of cultivated *C. annuum* has erect, small fruit (about 1 cm in length) that are pungent, red colored, deciduous and soft-fleshed. These traits promote consumption and seed dispersal by birds rather than mammals as birds do not have receptors for capsaicin, the source of pungency [10]. Through domestication and subsequent commercialization several domestication-related traits have been selected for, including compact architecture, increased efficiency of self-pollination and fruit set, early flowering and non-deciduous, pendant fruits. The diversity of uses for peppers has led to the development of individual *C. annuum* lines that have been selected for specific sets of consumer-driven fruit traits such as degree of pungency, flavor, color, shape, fruit wall thickness and drying ability [1]. It has been proposed that, in general, continued selection during domestication has led to lines with larger, non-pungent fruit with greater shape variation and tremendous increases in fruit mass [11]. These large-fruited non-pungent Bell or blocky type peppers were found in pre-Columbian Mexico and were first described approximately 500 years ago. The putative acyletransferase AT3, encoded by *Pun1,* is the primary determinant of pungency and non-pungent *C. annuum* share a common deletion in *Pun1* [12]. Even though the pungent pepper lines are considered the most important spice crop worldwide the large, non-pungent Bell peppers, also consumed worldwide, are the most economically important pepper type [1].

Breeding programs for crop improvement are enabled by the availability and detailed characterization of genetically diverse germplasm. A limited horticultural classification of pepper types with 7 main catagories and a total of 13 groups by fruit type, was developed to distinguish commercially significant varieties by Smith in 1987 [13]. This scheme is still relevant today as the only major change has been the division of the Small hot group into multiple categories by Bosland [1]. Standardized definitions of *Capsicum* descriptors were developed by the International Plant Genetic Resources Institute (IPGRI) and the United States Department of Agriculture, Germplasm Resources Information Network (GRIN) [14]. The IPGRI descriptors include 79 phenotypic traits divided into 25 vegetative, 16 inflorescence, 22 fruit, 6 seed and 10 yield and quality characters. However, the IPGRI descriptor for overall fruit shape is broadly defined by only 6 categories; Elongate, Almost round, Triangular, Campanulate,Blocky and Other. The USDA GRIN evaluation of peppers includes 55 descriptors including 5 growth, 38 morphology, 1 phenology, 3 chemical and 8 production traits. The 7 GRIN fruit shape descriptors; Elongate, Oblate, Round, Conic, Campanulate, Bell and Mixed are similar to those of IPGRI. GRIN includes as an additional trait 13 commercial categories that differ considerably from Smith's original groupings. Substantial *Capsicum* germplasm collections are held in several countries with over 53,000 total accessions held worldwide reported by the UN-FAO [15]. Some gene bank accessions have been characterized phenotypically with subsets of the IPGRI or GRIN descriptors [14]. The challenge in developing a practical phenotypic classification scheme underscores the added value of molecular characterization for understanding diversity.

The diploid *Capsicum* genome consists of n = 12 chromosomes with an estimated haploid genome size of 3.3–3.6 Gb [16], [17]. Molecular studies assessing the overall diversity of *C. annuum* breeding germplasm have been carried out using tens to a small number (<200) of low throughput, mostly anonymous markers such as RFLPs, AFLPs and SSRs [18]–[24]. Genome-wide molecular characterization of available germplasm enables identification of novel alleles and subsequent introgression via molecular breeding; differentiation of cultivars and classifying inbred lines into heterotic groups; development of core collections by identifying gaps and redundancy in germplasm collections; and monitoring genetic shifts that have occurred during domestication, breeding, regeneration and germplasm conservation [25].

With recent advances in sequencing technologies, cost-effective means for developing genome-wide functional markers have become available. Single nucleotide polymorphisms (SNPs) have become the marker of choice due to their abundance and uniform distribution throughout the genome [26]. Characterization of genomes and populations via the application of high-throughput SNP marker technologies have broadened the possibilities for breeding strategies from simply inherited trait integration using marker assisted selection (MAS) to genome-wide association studies (GWAS) and multi-locus trait integration using genomic selection (GS) [27]–[29]. These advances are expected to improve breeding strategies for complex traits such as the improvement of crop yield and resistance to biotic and abiotic stress [30].

A requirement for GWAS and GS-directed breeding strategies is the determination of genetic relatedness, the presence of population structure and linkage disequilibrium. The widely used methods for determining population structure, the Bayesian cluster estimation of population structure and LD implemented in the program Structure and principle components analysis (PCA), each analyze single mutations individually. Although it has been suggested that the accuracy of these methods may be compromised when analyzing large numbers of genome-wide markers in which there will inevitably be linkage, the addition of the admixture and linkage models to the Structure package have been useful for resolving complex populations and large datasets with linked markers [31]. Recent studies in tomato and potato have shown that these methods are complementary and biologically informative [32], [33].

DNA microarrays, initially used for expression analysis, have been used widely for both marker discovery and assays for known SNP polymorphisms [26]. Microarrays designed specifically for polymorphism detection have short, 25-nucleotide, probes (also known as features or oligos) and are able to detect sequence polymorphisms, termed single feature polymorphisms or single position polymorphisms (SFPs or SPPs), with good specificity [34]–[40]. Recently, this technology has been refined for use in the more complex genome of lettuce (*Lactuca sativa*) where a comprehensive analysis of lettuce diversity was defined across 5 species [40].

To more thoroughly understand *C. annuum* genomic diversity and improve breeding resources, a high-throughput marker discovery platform was designed for *Capsicum* [40]. A large collection of EST sequences [41], *Capsicum* GenBank sequences and conserved orthologous sequences [42], [43] were assembled into 30,815 unigenes that were used to design the Pepper GeneChip. In this paper we describe simultaneous detection of polymorphism in DNA using the Pepper GeneChip from a diversity panel of 43 pepper lines including 40 *C. annuum* lines and one line each of *C. frutescens*, *C. chinense* and *C. pubescens*. With this approach, over 30,000 robust markers were identified among the diversity panel including highly informative markers within both the 21 pungent and 19 non-pungent *C. annuum* lines. The dataset provided sufficient markers for assessing differences in allele frequencies around the *Pun1* locus between pungent and non-pungent lines. We also report the first high density genome-wide analysis of molecular diversity among *C. annuum* lines using gene based markers which identified genetic classes that were clearly and consistently defined.

## Methods

### Pepper GeneChip design

As described by Stoffel et al. [40], an array design which utilizes an Affymetrix GeneChip array format 49 with a 5 µm feature size and a maximum capacity ∼6.5 M features of 25 nt was used. Sequences submitted to Affymetrix for probe design included 31,196 unigenes consisting of 30,500 pepper unigenes assembled from EST sequences, 54 pepper promoter sequences and 642 COSII genes [43], which will be referred to here as the Pepper Chip assembly, (Figure S1, Dataset S1) [44]. Features were subdivided as follows: (1) 6,473,556 genomic tiling probes, (2) 24,336 control probes including 16,900 *Capsicum* probes in 13×13 blocks and B2 Affymetrix control probes surrounding each control block and (3) 33,886 anti-genomic probes for detecting background hybridization. Anti-genomic (AG) probes represent probes selected by Affymetrix that do not match any sequence in GenBank (in 2006) with G/C content (the number of guanines and/or cytosines) ranging from 5 to 18 per 25 nt probe (Figure S2B).

Probes were arranged to assay a 2 bp tiling path where possible. An Affymetrix quality score >0.25 and redundancy ≤3 were required [45], resulting in over 90% of probes having a G/C content between 7 and 14 (Figure S2A). Final probe reduction to 6,473,556 tiling probes was achieved by requiring unigenes to be covered by 10 or more probes and trimming 19.92% of probes for each unigene covered by 500 to 1000 probes, 8.96% from both the 5′ and 3' ends. A total of 30,815 unigenes were represented on the chip.

### Germplasm and DNA extraction

A set of 43 *Capsicum* lines representing four cultivated species (40 *C. annuum*, 1 *C. frutescens*, 1 *C. chinense*, 1 *C. pubescens*) were provided by I. Paran, The Volcani Center, Bet Dagan, Israel; P. Bosland, New Mexico State University, Las Cruces, N.M., USA; J. Prince, California State University Fresno, Fresno, CA, USA; Molly Jahn, Cornell University, Ithaca, NY, USA; Deruiter Seeds, Enza Zaden; Monsanto; Nunhems; Rijk Zwaan; Syngenta and Vilmorin. Seeds were germinated in a glasshouse under standard conditions for *Capsicum* [46]. DNA was extracted from 2.0 grams of developing leaves, up to 3.0 cm in length. Leaves were collected from 2 to 6 individuals for each line, flash frozen in liquid nitrogen and stored at −80°C. A modified CTAB procedure was used for genomic DNA extraction [40]. Genomic DNA was quantified by agarose gel electrophoresis with a lambda DNA standard.

### Chip hybridization

For each hybridization, 30 ug total genomic DNA was fragmented to 50–250 bp and visualized by agarose gel electrophoresis. Following fragmentation, DNA was end-labeled and hybridized to the Pepper GeneChip as described [40]. Three replicate hybridizations were carried out for each line. Hybridization levels were adjusted by correcting for background followed by quantile normalization across all chips. To test for consistency of replicate hybridizations, a cluster analysis was performed in R on normalized hybridization values across all chips using 5,431 probes covering known polymorphisms. Close clustering of all three replicate chips for each variety was required prior to SPP analysis (Figure S3). The effect of G/C content on hybridization efficiency was determined (Figure S2C). There was no difference in the percentage of probes above background between lines. Of tiling probes, 76% were hybridized at levels above background with probes having between 9 and 13 Gs and Cs having the best performance.

### SPP detection

Samples were analyzed based on SPPdev values using an algorithm designed for polymorphism detection across multiple genotypes simultaneously (RIL algorithm) as described by Stoffel et al. [40]. Probe hybridization values were weighted using an empirically-determined weighting factor for pepper based on sensitivity of bases within an oligo to the position of sequence polymorphisms (Figure S2D). The SPPdev ratio is a measure of the hybridization difference between modes of a bimodal distribution of SPPdev values for a given position across all chips. For each polymorphic position, an allele call (A, B or -) was assigned for each chip. When there was a difference in the allele assignment between the 3 replicate chips of a given line, the summarized allele assignment was designated as inconsistent (I). Thus, the 3 replicates were summarized as A, B, C, D, I or “–”as follows: A/A/A = A; B/B/B = B; B/B/-  = C (not A); A/A/-  =  D (not B); -/-/-  =  “-“ and A/A/B, B/B/A, A/B/-, -/-/A or -/-/B  =  I. The pepper SPP detection software package and its manual can be downloaded at https://pepper.ucdavis.edu/public/data.php.

The RIL algorithm allows the stringency for calling SPPs to be increased by increasing minimum SPPdev ratio, and minimum number of probes above background (informative probes) required for SPP detection. The dataset can be further filtered by requirements for minimum number of bases spanned by an SPP, minimum and maximum allele frequencies allowed and maximum inconsistent (I) and missing (-) calls allowed across all genotypes. A schematic summarizing the collection and processing of data is presented in Figure S4.

### Validation

Using BWA [47] and SAMtools [48] a set of high quality SNPs, heterozygous positions, and InDels were identified in Illumina GA2 (IGA) transcriptome sequence assemblies derived from root, leaf, flower and several fruit tissues of Early Jalapeño, CM334 and Maor. Custom Perl scripts were used to identify false positive SPPs by comparison to the IGA assemblies and SPP markers mapped in two pepper mapping populations. A SPP was considered a true positive if SNPs were within 8 bp of the SPP ranges. An 8 bp range on either side of detected SPPs was chosen to account for detection of SPPs with the overlapping 25 nt oligo design and empirically determined sensitivity of oligos (Figure S2D). SPPs that were not represented by IGA sequence were excluded from the validation as we were not able to verify if they were true or false SPPs. In order to determine the effect of adjusting detection stringency and filtering parameters on data quality, SPP detection rates and false discovery rates (FDR) were compared between resulting datasets (Figure S5). Datasets generated at SPPdev ratio settings of 1.2, 1.5 and 2.0 are available in Datasets S3, S4 and S5 respectively.

In order to determine if sequences surrounding an apparent SPP were present in multiple unigenes (multi-copy sequences), each SPP plus and minus 8 bases on either side of the SPP range was queried using BLASTn [49] against two separate search sets of sequences: the pepper whole IGA assembly and the EST assembly that was used for the array design. To maintain confidence in the BLAST hits a minimum of 95% of the subject in the query with no more than two mismatches was required.

### Accuracy of calls

Since most of the sequences used for the Pepper Chip assembly were derived from a F_1_ hybrid pepper variety (Bukang), we were able to design and run SNP assays (Kbiosciences, Hoddesdon, UK) from our assembly for all lines. We determined the correlation between calls made by the SNP assay and SPPs spanning the SNP nucleotide for 27 polymorphisms across the 43 lines for a total of 1,161 allele calls (Table S2).

### Analysis of *C. annuum* diversity

The dataset used for diversity analysis was generated with minimums of SPPdev ratio 1.2, two informative probes and four bases spanned. With these parameters, 104,470 SPPs were identified within 23,724 unigenes. This dataset was filtered by removing SPPs within multi-copy sequences (homology to multiple assembled sequences). C and D calls were converted to B and A, respectively. Then requirements of minimum A or B allele frequency greater than or equal to 0.02 (1 of the 43 genotypes in the panel), zero inconsistent calls and no missing values were applied resulting in a final dataset of 33,401 SPPs/13,323 unigenes with an estimated SPP FDR of 6.8% (Dataset S2, Figure S5E).

A phylogeny of the *C. annuum* lines was inferred using the PHYLIP 3.69 package [50]. SPPs polymorphic among the 40 *C. annuum*, the *C. frutescens* and *C. chinense* lines (leaving out *C. pubescens*-specific SPPs) were selected. Unique SPP allele profiles (haplotypes) across the panel for each unigene were selected to reduce linked markers showing no recombination across the panel. The SPP markers were treated as restriction site markers, converted from A and B to 1 and 0. The seqboot module was used to create 7,500 re-sampled data sets for bootstrapping. The restdist package using the modified Nei and Li method was used to generate distance matrices for input into the Fitch module for tree building. The Fitch-Margoliash distance method with global rearrangement and randomized input order of species with 5 jumbles was used to generate each of 7,500 replicate trees. The consensus tree with bootstrap values was calculated and visualized using MEGA4 [51] with *C. frutescens* & *C. chinense* used as the outgroup root.

### Population structure analysis

The Bayesian cluster estimation of population structure was carried out using the software Structure [31]. SPPs polymorphic within the *C. annuum* lines identified within unigenes that were also mapped in a *C. frutescens* × *C. annuum* (FA) RIL population (Van Deynze unpublished) were selected and map positions based on the FA map were assigned to each SPP. Among the resulting set of SPPs with associated map positions, unique allele profiles across *C. annuum* lines for each genetic bin were selected to eliminate markers completely linked across the panel. Ten replicates were performed for each defined value of K number of clusters assumed from K = 1 to K = 10 using the linkage model and independent allele frequencies model [52]. A constant value of lambda (allele frequencies parameter) was defined at K = 1 to be 0.5397 and this value was used for all subsequent runs at values of K = 2 to 10. Each run used an admixture burn-in period 35,000 iterations then 35,000 burn-in iterations followed by 30,000 Markov Chain Monte Carlo (MCMC) iterations. The replicate producing an output with the highest probability for each K value was selected.

### Principal component analysis

For principle component analysis, 6,426 SPP markers polymorphic among the 40 *C. annuum* lines were used. These SPPs were found within 3,818 unigenes and converted to unigene haplotype frequencies using custom Perl scripts [40]. Frequencies were calculated as the number of times the haplotype occurs in the panel divided by the total number of genotypes in the panel. Haplotype frequencies were used as input for principle component analysis using the SAS/STAT^®^ software's PRINCOMP procedure (Version 9.1.3, SAS Institute, Cary, USA). Eigenvalues for each of the first three principal components were extracted for each genotype, visualized by 3-dimentional graphs and used to cluster lines using the Ward method. Each of the first three Eigenvalues were tested for separation of groups defined by either breeding class or Structure clusters using an analysis of variance (ANOVA) and pairwise Student's T-tests for means separation. Graphical and statistical analyses were performed using JMP (JMP, Version 7. SAS Institute Inc., Cary, USA).

### Genetic diversity estimates

Genetic diversity (H_e_) was estimated from the 6,426 *C. annuum* polymorphic SPP markers using PowerMarker [53]. H_e_ is defined as the probability that two randomly chosen alleles from a population are different [54].

## Results

### Affymetrix Pepper GeneChip design

A high-density pepper genotyping array was created based on the design of the Lettuce Affymetrix GeneChip [40]. The pepper array includes 33,886 features for anti-genomic (AG) background probes and 6,473,556 features for genomic tiling probes. Probe selection resulted in 30,815 unigenes represented. Coverage ranged from 11 to 1,597 probes per unigene with 80% of the unigenes covered by 70 to 375 probes (Figure S1B).

### SPP detection, false discovery and accuracy

Forty-three *Capsicum* lines were hybridized to the Affymetrix Pepper GeneChip for simultaneous polymorphism detection (Table 1). Large changes in the number of SPPs identified and a significant effect on FDR was observed with changing the parameters minimum SPPdev ratio, minimum bases spanned and inconsistent calls allowed (Figure S5, Table S1). To determine accuracy of calls, allele calls between SPP and SNP assays were compared. A high correlation of allele calls was observed, with an exact match between the SNP and SPP assays for 95% of all alleles called and 99.9% of all unambiguous calls made by both assays (Table S2). The SNP assay detected a heterozygosity rate of 3.5% across the 43 lines.

**Table 1:** Pepper lines.

Classification^a^	Fruit Shape^b^	Name	Pungency^c^	Unique	Unique	
				SPP alleles^d^	SPP alleles^e^	
*C. annuum* var. *annuum*						
Bell	Blocky	Yolo Wonder	np	0	0	
	Blocky	Rumba	np	1	1	
	Blocky	Violetta	np	2	2	
	Blocky	Bruinsma Wonder	np	7	14	
	Blocky	Maor	np	9	10	
	Blocky	Ariane	np	7	8	
	Blocky	Charleston Belle	np	1	2	
	Blocky	Jupiter	np	0	0	
	Blocky	King of the North	np	3	3	
	Blocky	Dempsey	np	3	3	
	Blocky	Infante	np	17	20	
	Blocky	Grande de Reus	np	11	13	
	Blocky	Grosso de Nocera	np	3	3	
	Triangular	Dolmalik	np	2	2	
Long Wax	Triangular	Midal	np	30	44	
	Triangular	Sweet Banana	np	6	9	
	Triangular	Long Yellow Marconi	np	46	58	
	Triangular	Corno di Toro	np	12	16	
Other	Triangular	Doux des Landes^1^	np	19	29	
	Other	Carre d'Asti^1^	p	15	16	
	Elongate	Lange Westlandse Rode^1^	p	69	87	
	Elongate	Sivri Biber^1^	p	19	25	
	Elongate	Erjintiao^2^	p	121	162	
	Elongate	Jeju^2^	p	19	29	
	Elongate	Cheongsong^2^	p	76	111	
	Elongate	Milyang^2^	p	123	159	
Cayenne	Elongate	Carolina Cayenne	p	115	210	
Anaheim chili	Triangular	NuMex R Naky	p	30	49	
	Triangular	NuMex Joe E Parker	p	15	19	
Jalapeño	Elongate	Jalapeño-M	p	19	22	
	Elongate	Early Jalapeño	p	13	23	
Ancho	Triangular	Ancho 101	p	119	144	
Small hot	Elongate	CM 334	p	205	330	
	Elongate	PI 201234	p	110	163	
	Elongate	Charleston	p	21	33	
	Elongate	Perennial	p	53	73	
	Elongate	Pusa Jwala	p	121	170	
	Elongate	G-4	p	87	126	
	Elongate	PSP-11	p	53	69	
	Elongate	Thai Bird	p	85	129	
*C. chinense*	Elongate	PI 159234	p	4960		
*C. frutescens*	Almost Round	2814-6	p	3859		
*C. pubescens*	Almost Round	Rocoto	p	13643		
Total Unique SPPs				24129	2386	

a Classification of *C. annuum* lines assigned according to Smith [13]

b Fruit Shape of glasshouse grown fruit as defined by IPGRI, AVRDC and CATIE [14]

c Pungency is indicated by np (non-pungent) and p (pungent)

d Unique SPP alleles among all 43 lines (33401 total SPPs)

e Unique SPP alleles among 40 *C. annuum* lines (6426 total SPPs)

1 Other classification, European origin

2 Other classification, East Asian origin

### Effect of copy number variation on SPP calls at the *Pun1* locus

**Figure 1:**
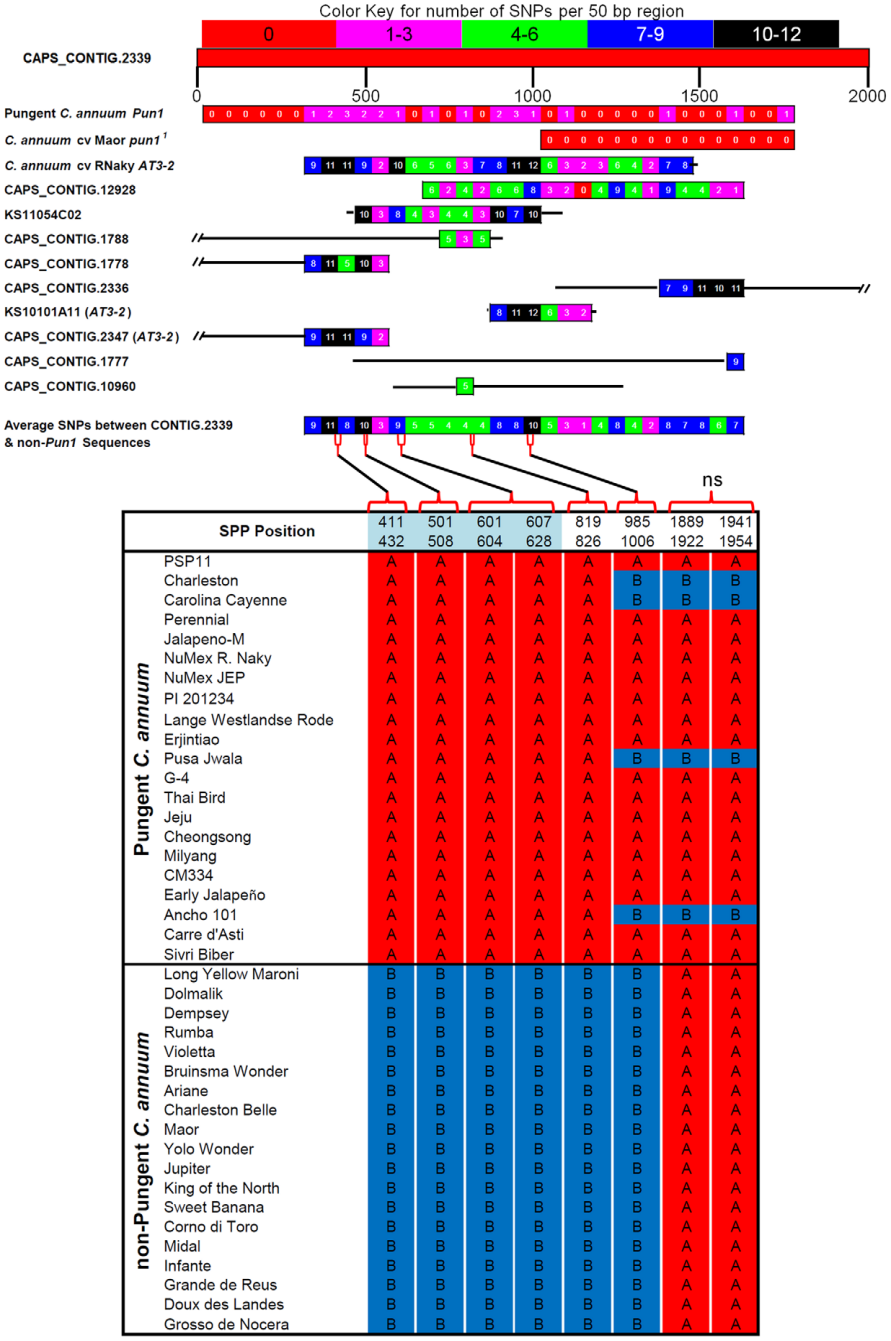
SPPs identified in the *Pun1* locus (Pepper GeneChip CAPS_Contig.2339). At the top, the diagram represents an alignment of CAPS_Contig.2339 with GenBank sequences for *Pun1*(FJ755173.1, GU300812.1, AY819028.1, AY819029.1, AY819032.1, AB206919.1), *pun1^1^* which has a large 5' deletion (gb AY819031.1), *AT3-2* (FJ687524.1) and Pepper Chip assembly unigenes with significant similarity (>80% identity, >50 nucleotides aligned). The number of SNPs per 50 bp window between CAPS_Contig.2339 and aligned sequences are indicated by color boxes with key shown above. Regions not aligning are indicated by black lines. Below are the (40, option1 or 43 option 2) *C. annuum* lines with SPP calls (red  =  A, blue  =  B) along the length of the CAPS_CONTIG.2339 from 5' to 3'shown left to right with positions shown above the allele calls. Black lines link the positions of the SPP calls to the alignment cartoon. SPPs at positions with no additional sequence information are indicated (ns).SPP positions, indicated in the top row of SPP calls, highlighted in blue were identified as duplicated sequences among the pepper assemblies and thus were removed prior to subsequent analyses.

To investigate further the source of false positives, the potential effects of copy number variation and paralogs on SPP calling was examined. *Pun1*, formerly known as *C*, is the primary determinant of pungency in *Capsicum* and encodes the putative acyltransferase AT3 that is required for the synthesis of capsaicin [12]. *Pun1* corresponded to oligonucleotides derived from CAPS_CONTIG.2339 on the Pepper Chip. *C. annuum* lines carrying the null *pun1^1^* allele, which has a deletion of the 5' coding region, are non-pungent. Lines were classified by the presence of the *Pun1*, pungent, versus *pun1^1^*, non-pungent, alleles (Figure S7, Table 1). For a large deletion such as in *pun1^1^*, there should be no informative probes for SPP detection in non-pungent lines. However, SPPs were detected across the deleted region, likely due to hybridization of paralogous acyl-transferases with shared sequence identity to CAPS_CONTIG.2339 probes (Figure 1). A pseudogene, *AT3-2,* present in *Capsicum* genomes is annotated in GeneBank and has 84% DNA sequence identity to a portion of the *Pun1*coding region. In addition, nine unigenes with significant identity to CAPS_CONTIG.2339 were identified in the Pepper Chip assembly. Two unigenes shared 100% identity with *AT3-2* leaving 7 additional unigenes with significant similarity to *Pun1* (Figure 1). Sequences for *Pun1, pun1^1^*, *AT3-2* and Pepper Chip assembly putative acyltransferase unigenes were aligned with CAPS_CONTIG.2339 revealing common regions that were highly conserved (87–98% identity) or moderately conserved (75–85% identity) (Figure 1). Six SPPs were detected in the deleted region of *pun1^1^*, of which five had allele calls specific to pungent (A, high hybridization) and non-pungent (B, low hybridization) lines. Five SPPs overlapped with regions less conserved between *Pun1* and the putative acyltransferase unigenes. At the 3' end of the *pun1^1^* deletion, GenBank sequences indicate several SNPs between pungent lines. This region had lower conservation between aclytransferase unigenes and CAPS_CONTIG.2339. SPPs detected in this region had marker profiles among the pungent lines similar to profiles 3' of the *pun1^1^* deletion suggesting that these calls are accurate and there is an additional *Pun1* allele with multiple 3' polymorphisms in four of the 21 pungent lines.

Several SPPs identified within the *pun1^1^* deletion are detecting polymorphisms between paralogs and therefore could be considered false positives as they indicate SNPs or Indels not a large deletion. To verify the influence of duplicated sequences on false positive rates, BLAST searches were carried out with SPPs and surrounding nucleotides against pepper transcriptome assemblies to identify SPPs found in sequences represented multiple times in the transcriptome. The removal of SPPs with multiple significant BLAST hits caused a reduction in the false positive rate of 1% to 1.4% across datasets (Figure S5E). Taken together, this indicates that there is meaningful information in SPP calls within multi-copy sequences. However, the information is complex, difficult to interpret and may be observed as false positives without the benefit of sequence data. Therefore, SPPs identified within multi-copy sequences were removed from further analyses.

### Allele sharing around *Pun1^1^*

The 43 *Capsicum* lines in this study included 40 *C. annuum* lines and a representative from each of three additional cultivated species *C. frutescens*, *C. chinense*, and *C. pubescens* (Table 1). The 40 *C. annuum* lines, 21 pungent and 19 non-pungent, were selected by breeders specifically to represent a broad range of germplasm currently used in commercial breeding programs. A total of 33,401 polymorphic SPP markers within 13,323 unigenes were identified across the 43 lines (Dataset S2). Within this set, 6,426 SPPs covering 3,818 unigenes were polymorphic among the 40 *C. annuum* lines.

**Figure 2:**
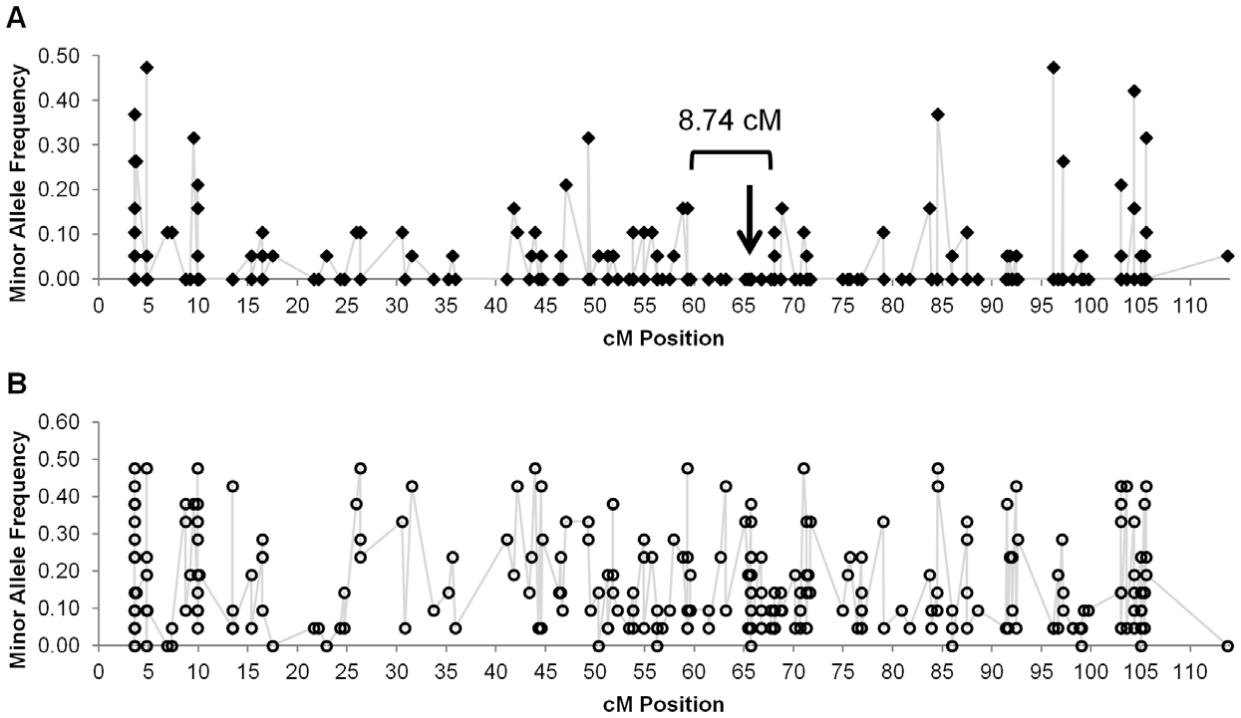
Polymorphism among pungent and non-pungent lines on chromosome P2. Minor allele frequency by cM position across (A) non-pungent and (B) pungent lines. An 8.75 cM region of P2 containing *pun1^1^* (**↓**) is monomorphic among the non-pungent lines.

A total of 276 *C. annuum* SPP markers have been mapped to linkage group P2, including *Pun1* (CAPS_CONTIG.2339) at position 65.7 cM (Figure 2A). Among the 19 non-pungent lines in this study, there were 42 monomorphic SPPs within 34 unigenes around *pun1^1^* that extended over 8.74 cM of P2. All but one of these SPP markers were polymorphic among the 21 pungent lines with varying allele frequencies (Figure 2B). Of the nine unigenes with significant similarity to *Pun1,* seven mapped to the same position as *Pun1* while the remaining two were not mapped. A search against the tomato genome (ITAG2.3, http://solgenomics.net/) shows that there are 3 acyltransferases highly similar to *Pun1* (< e-115) present in tandem on T2 and a total of 5 sequences annotated as acyltransferase sequences between positions 40,149,035 and 40,193,345 of T2.

### Distribution of allele frequencies and polymorphism within and between groups

**Figure 3:**
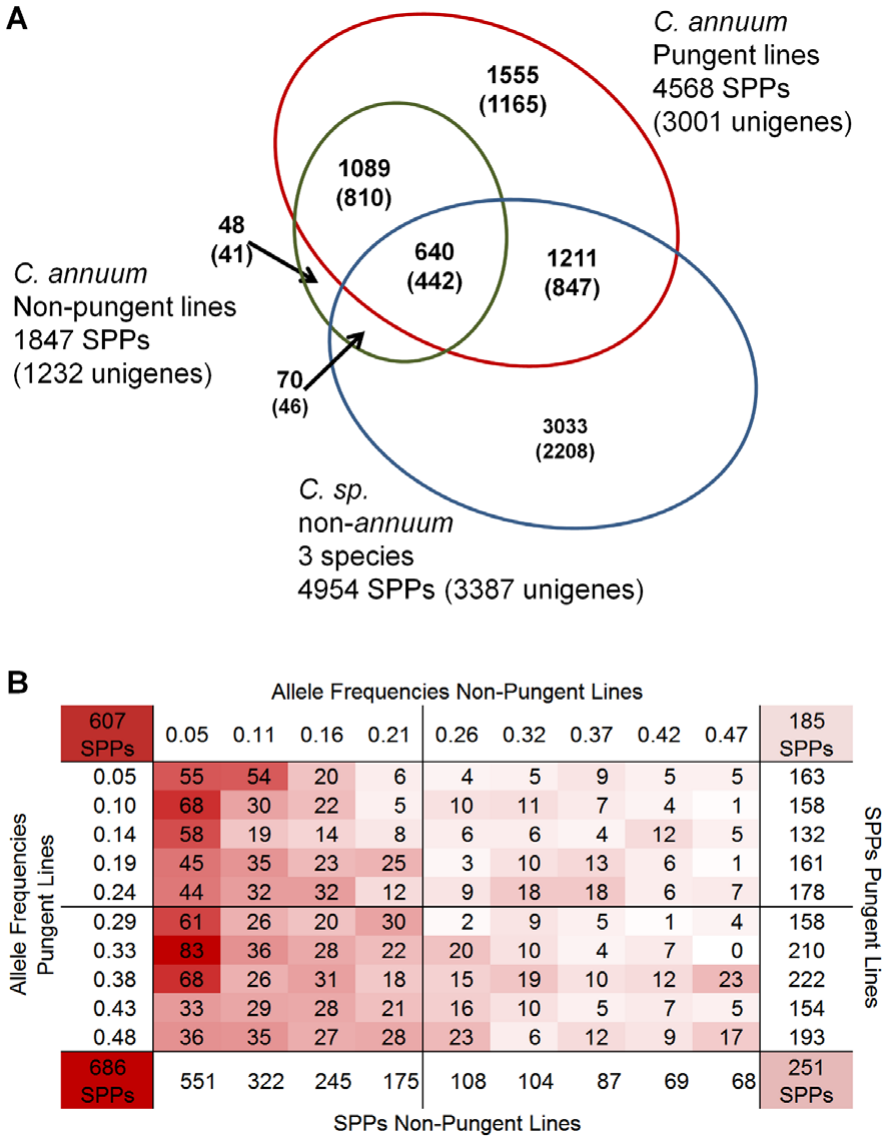
Polymorphisms among 19 non-pungent, 21 pungent *C. annuum* and 3 non-*annuum* lines. (A) Venn diagram depicting the number of SPPs and unigenes (shown in parentheses) polymorphic within each group out of 9,272 SPPs within 5,712 unigenes having >2 minor alleles across the 43 lines. (B) Allele frequency matrix for 1729 informative SPPs among pungent and non-pungent *C. annuum* lines. Numbers indicate SPPs found at each minor allele frequency pair. At the corners are shown the total number of SPPs for minor allele frequencies pairs divided by 0<0.25 and 0.25<0.5. At left and bottom are shown total SPPs at each minor allele frequency for pungent and non-pungent lines respectively.

Of the 33,401 polymorphic markers among the 43 lines, the overall distribution of SPPs with unique alleles was highest in the non-*annuum* lines (Table 1), with the largest number of unique alleles identified in *C. pubescens* followed by *C. chinense* and *C. frutescens* at 13,643, 4,960 and 3,859 SPPs respectively. The increase in SPPs with the inclusion of non-*annuum* lines was largely but not solely due to these unique alleles with over 3,000 SPPs having alleles specific to two of the three non-*annuum* lines and over 1,400 having alleles specific to all three non-*annuum* lines (Figure 3A).

Of the 6,426 SPP markers polymorphic within the *C. annuum* lines, 1,667 had an allele unique to 1 variety across all 43 lines and 2,386 across the 40 *C. annuum lines*. The overall distribution of unique alleles was generally greater in pungent versus non-pungent lines (Table 1). While only 0 to 46 unique alleles were identified among non-pungent lines, unique alleles among pungent lines ranged from 13 to 205 with the largest number of unique alleles identified in the semi-domesticated land race CM334. Among the SPP markers polymorphic within *C. annuum* lines having greater than 1 minor allele (across all 43 lines), there were over two times more polymorphic SPPs within the pungent (4,568) then the non-pungent (1,847) lines (Figure 3A). Over 90% (1,729) of the markers polymorphic in the non-pungent group were also polymorphic among pungent lines. Of these 1,729 markers, the distribution of minor allele frequencies was highly skewed toward low frequencies among the non-pungent lines while more evenly distributed among the pungent lines (Figure 3B, Dataset S6). Even so, 251 highly informative markers (>0.25 minor allele frequency) across both groups were identified. Genetic diversity is significantly reduced (p<0.0001) in non-pungent vs pungent *C. annuum* lines with estimated H_e_ values of 0.07 and 0.23 (0.22 without CM334 and PI 201234 landraces included in pungent population), respectively.

### *C. annuum* diversity and population structure

**Figure 4:**
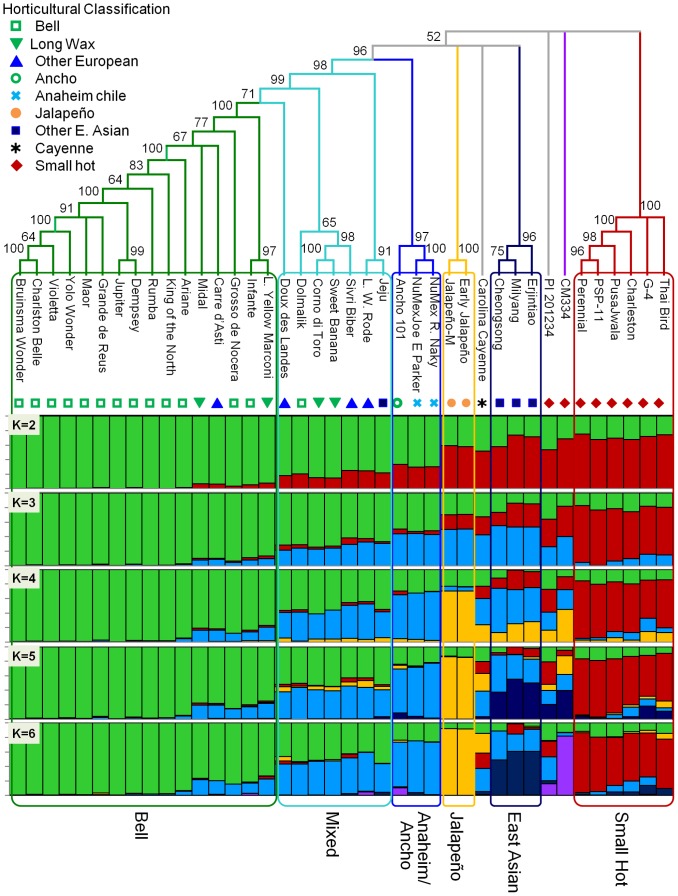
Consensus tree and population substructure estimated from SPP markers. At the top, a Fitch & Margoliash tree of 40 *C. annuum* lines, rooted with *C. frutescens* and *C. Chinense*. The majority-rule consensus cladogram (overall equal branch lengths) was generated from 13,621 SPP markers. Numbers associated with branches indicate percent support based on 7,500 bootstrap replicates. Branches with less than 50% support have been collapsed. At the bottom is shown *C. annuum* population substructure determined using Structure with 2,712 mapped SPP markers for K = 2 to K = 6. Each genotype is represented by a vertical column and genotypes are ordered according to the cladogram. Each color bar represents a different subpopulation and the proportion of a given variety's color bar represents the proportion that variety belongs to the corresponding subpopulation. The branches of the cladogram are colored according to the highest proportion subpopulation assignment when K = 6 with grey branches indicating highly admixed individuals, having no more than a 0.60 fraction assigned to any subpopulation. Grouping by common structure subpopulation constitution is indicated by colored border with assigned names shown below. Long Yellow Marconi and Lange Westlandse Rode are abbreviated L. Yellow Marconi and L. W. Rode, respectively.

**Figure 5:**
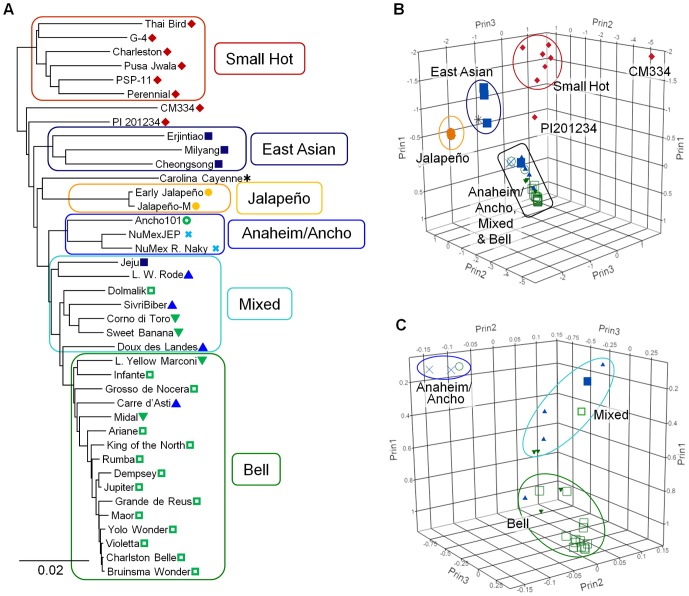
Principle component analysis & phylogram derived from SPP markers. (A) A representative Fitch and Margoliash phylogram with branch lengths reflecting phylogenetic distances based on Nei and Li genetic distances. Boxed clades and labels correspond to clusters based on Structure simulation at K = 6. Long Yellow Marconi and Lange Westlandse Rode are abbreviated L. Yellow Marconi and L. W. Rode, respectively. (B) Graph showing the coordinates of the first 3 principle components for each variety. Clusters are circled and labeled. Non-clustered lines PI 201234 and CM334 are labeled separately. In this view the Anaheim/Ancho, Mixed and Bell clusters, boxed in black, are difficult discriminate, therefore, this area was expanded in (C) which shows the arrangement of these clusters. Symbols correspond to those representing horticultural classifications in Figure 4.

Phylogenetic trees were constructed using 13,621 SPP markers found to have unique allele profiles within each unigene among lines. Using the *C. frutescens* and *C. chinense* lines as an outgroup to the *C. annuum*, the bootstrap consensus cladogram showed a general clustering of horticultural classes (Figure 4). Clades representing several pungent types including Small hot, Other-East Asian, Jalapeño and Anaheim plus Ancho were well supported, at 96% to100%. Carolina Cayenne and the *Phytophthora*-resistant Mexican land races CM334 and PI 201234 were each found on independent branches among the pungent clades. The relationships between the pungent clades were unresolved. The remaining lines form a well-supported (98%) monophyletic grade sister with 96% support to the pungent Anaheim/Ancho clade. The non-pungent Bell types were found in the most derived positions (Figure 5A).

A model-based clustering algorithm, implemented in the software Structure, was used as a second means of identifying subgroups among the *C. annuum* lines (Figure 4). The algorithm identifies a predetermined number of clusters, K, that have distinct allele frequencies. A genome can have membership in multiple clusters with genomic membership coefficients summing to 1. The Structure algorithm was run using 2,712 mapped SPP markers with K values set at K = 2 to K = 10. At K = 2 there is a varying degree of admixture across the lines. The Structure output was arranged by line according to the phylogenetic tree. At K = 2, the two clusters are anchored by the pungent Small hot and the non-pungent Bell types with a clear transition in membership coefficients seen where the Anaheim/Ancho clade branches off from the rest of the pungent groups in the phylogeny, at the base of the monophyletic grade leading to the Bells. The Bell cluster contributes to the genome of all individuals across all values of K with the exception of Milyang at K = 6. Each increase in K up to K = 6 splits one of the clusters obtained with the previous value with at least one individual having >0.60 membership to the new cluster. At K = 7 and greater no individuals were assigned to any additional cluster at >0.50 membership. Therefore, K = 6 was chosen as the appropriate K value for clustering of subpopulations. Of the resulting 6 clusters, 5 correlated with clades in the phylogeny with 96% or greater bootstrap support. The sixth cluster which only included one variety, the Mexican land race CM334, was found on an independent branch in the cladogram. There are seven pungent and non-pungent lines, while having less than 0.60 membership in any given cluster, share similar membership coefficients for both the Bell and Anaheim/Ancho clusters indicating a seventh cluster by common overall genomic constitution and will be referred to as the Mixed cluster. These are present in the phylogeny at the basal portion of the monophyletic grade leading to the derived Bell types. There were two additional lines, the *Phytophthora* resistant accession PI201234, and Carolina Cayenne, that showed a high degree of unique admixture, consistent with their positions in the phylogenetic tree.

A third method employed to examine genetic diversity and substructure within the *C. annuum* lines was a Principal Components Analysis (PCA) of allele frequencies across the 40 lines for 3,818 polymorphic unigene haplotypes. Cumulatively, the first three principal components (PCs) explained 27% of the variation in the data with 12%, 8% and 7% by PC1, PC2 and PC3 respectively. A 3-dimentional graphical representation of the first three Eigenvalues for each line indicates clusters that correspond to the clusters identified by structure and phylogenetic analyses (Figures 5B, C and S6). The only exception is that the PCA placed Carolina Cayenne with a cluster including the East Asian cluster while this line is unique in the Structure and phylogenetic analyses.

Analysis of variance indicated that the groups defined by the Structure clusters shown in Figure 4 were significantly different for all three PCs (P<0.0001). T-tests for mean separations between clusters showed that PC1 (P<0.05) and PC3 (P<0.0005) separated all groups. PC2 separated the Small Hot and East Asian clusters from all others (P<0.01) (Table S3).

### Hybridization efficiency of *Solanum spp*

To determine the potential utility of the Pepper GeneChip across *Solanum spp*., DNA hybridization efficiency for representative Solanum crops including eggplant (*S. melongea*), tomato (*S. pennellii* and *S. lycopersicum*) and potato (*S. tuberosum*) was determined. Hybridization efficiencies, measure as the number of probes above background (the 90^th^ percentile of anti-genomic probes) were ∼50% lower for each of the *Solanum* lines than for *Capsicum* (Table S4). However, greater than 99.9% of all unigenes were represented by probes above background for all of the *Solanum* lines tested, indicating the degree of homology between transcriptomes in *Solanum* species.

## Discussion

### Pepper GeneChip design

High throughput marker discovery and analysis of genetic diversity among crop species has become essential for modern breeding programs. In order to improve genomic and genetic resources for pepper breeding we have developed a method for high-throughput parallel detection of polymorphisms in pepper. Taking advantage of recent *Capsicum* EST sequencing efforts [41] along with a custom genotyping array design, hybridization methods and algorithms [40], an Affymetrix Pepper GeneChip was designed based on 30,815 pepper unigenes with an average of 210 probes per unigene. The utility of the array was demonstrated by its application in the identification and analysis of polymorphism across a diversity panel of 43 *Capsicum* lines and Solanaceae species.

### SPP detection and data filtering

The efficacy of the chip and custom algorithms for polymorphism detection were tested using a panel of 43 *Capsicum* lines. Replicate hybridizations of genomic DNA from each line were completely reproducible by cluster analysis. Filtering parameters for SPPs identified relevant criteria for filtering SPPdev ratios, number of informative probes, number of bases spanned missing calls, inconsistent calls and allele frequencies. Adjustment of these parameters resulted in datasets ranging from 20,000 to over 80,000 SPP markers identified within 10,000 to 20,000 unigenes across the 43 *Capsicum* lines at false positive rates ranging from 6.2% to 10.3%. The lowest FDR among the datasets analyzed was observed with a SPPdev ratio ≥1.5, bases spanned ≥4, zero inconsistent and zero missing calls allowed with multi-copy sequences removed with accuracy of calls at known SNPs of 99.9%. Similar results were observed in the lettuce diversity study, although FDR was ∼5% lower for lettuce, likely due to a more comprehensive set of mapped SPPs used for validation in lettuce [40]. This study defined genetic diversity in *Capsicum* using a set of reliable gene-based SPP markers with greater than 40 fold resolution than previously reported.

Presenting the results of adjusting filtering parameters enables users to choose an appropriate dataset based on the intended application. For analysis of diversity and population structure, a stringent dataset with maximum coverage is desirable. We chose minimum detection requirements of 1.2 SPPdev ratio, 2 informative probes and 4 bases spanned and eliminated inconsistent calls and SPPs within multi-copy sequences for analysis of lines in this study. A SPPdev ratio of 1.2 was selected since it provides 30% more SPPs, 13% more contigs, with a minimal increase, 0.6%, in FDR compared with a SPPdev ratio of 1.5. Allowing I calls can overestimate diversity by defining new haplotypes. Eliminating SPPs covering multi-copy sequences reduces potential ambiguity, as shown with (*Pun1*). For more line-specific applications, such as attempting to identify SPPs between two closely related lines, it may be more advantageous to use the low stringency (2 bases spanned) dataset at an SPPdev ratio of 1.5 and 0 I calls with over 83,000 SPPs in 21,000 unigenes and an estimated FDR of 9.7%.

The quality of the data observed for polymorphism detection with the Pepper GeneChip was comparable to the most current sequencing technologies. The custom design and analysis of array data produce 6 to 10% FDR while current FDR estimates for SNPs derived from sequencing data can range from 5 to 50% [55]. Similar to sequencing, several filters need be applied at the expense of eliminating true positives with microarray data. SNPs derived from sequenced populations have been filtered to remove rare alleles particularly unique alleles. We found the rare alleles identified by our method to make biological sense and did not observe a difference in FDR with the inclusion of unique alleles (data not shown). Sequence depth requirements for SNP discovery are analogous to our replicate chips and multiple probes per position requirements. Until recently, high-throughput transcriptome sequencing and subsequent data analysis was cost prohibitive for parallel high-throughput SNP discovery. Microarray based polymorphism discovery was an attractive alternative even though a small region surrounding a polymorphism rather than the precise nucleotide position of a polymorphism is identified. Very recent advances in sequencing technologies leading to next generation sequencing and beyond have led to more cost effective methods for high-throughput sequencing providing even larger and more detailed marker datasets. At the current time, the cost effectiveness of sequencing has surpassed microarray based methods for both polymorphism discovery and expression analyses. However, arrays (eg, Illumina Infinium and Affymetrix Axiom Arrays) remain an affordable method for SNP assays designed based on known SNPs.

### Polymorphism detection across 43 *Capsicum* lines

The capacity for polymorphism detection using the Pepper GeneChip was assessed using a diversity panel of 43 *Capsicum* lines. With 33,401 robust SPPs detected, 24,129 represented a unique allele across the panel. The three non-*annuum* lines had the largest number of unique alleles with diversity estimates congruent with other molecular studies that have shown *C. annuum* is most closely related to *C. frutescens* followed by *C. chinense* as a three-species complex, and more distantly related to *C. pubescens* [56], [57]. Among the 40 *C. annuum* lines, we identified 6,426 high quality SPP markers. As a whole, these markers indicate a 3-fold reduction in genetic diversity within non-pungent vs. pungent lines. Consistent with the derivation of the non-pungent lines from pungent precursors, >90% (1,729) of the non-pungent group polymorphic markers were also polymorphic within the pungent group. Among these, 251 markers were highly informative (>0.25 allele frequency) within both groups allowing for effective use for breeding. Inspection of allele frequencies between pungent and non-pungent lines across pepper chromosome P2 confirmed lower diversity in non-pungent lines. Additionally, allele distortion around *pun1^1^* is a dramatic 100% over >8 cM, indicating a common conserved region/introgression shared among these non-pungent lines. Thus, high-throughput marker discovery using the Pepper GeneChip has produced robust SPP markers providing high resolution diversity estimates, highly informative markers sets and new information regarding the genomic constitution of non-pungent *C. annuum*.

### *Capsicum* diversity and population structure among *C. annuum*

The use of a genome-wide set of markers specifically tailored for each of three independent methods; distance based phylogeny calculated using Fitch; a Bayesian analysis of population structure (Structure) and a principle components analysis (PCA) resolved a clear description of genomic contribution and relatedness between 40 *C. annuum* lines into six clusters. These were statistically supported by phylogenetic bootstrapping and t-tests on PCA principle components 1 and 3. All three analyses consistently identified six clusters of related genotypes and show a grade of reduced diversity in the non-pungent *C. annuum*. However, the phylogenetic relationships between the identified pungent clusters were unresolved and the genomic constitution of three pungent accessions found to be unique within the panel could be misinterpreted using only one or two of these methods, the results of these three analyses taken together provided a clearer understanding of the population substructure and relationships between lines.

The PCA results for PI201234 and Carolina Cayenne are difficult to interpret alone. PCA places PI201234 between the Anaheim/Ancho and Mixed groups and Carolina Cayenne with the Japanese/Korean types. However, relationships become clear with the benefit of the Structure and phylogenetic analyses. PI201234 and Carolina Cayenne, were each found to be highly admixed and genetically independent from the six identified Structure clusters with admixture components that resolve the PCA results. PI201234, while on a solitary branch in the phylogeny, has substantial contributions from the CM334, Anaheim/Ancho, Small hot and Bell clusters in the Structure analysis is in agreement with its position in the PCA. Similarly, the Structure analysis showed Carolina Cayenne having substantial contributions from the Mexican Hot, Small hot, and Anaheim/Ancho clusters and the PCA coordinates for Carolina Cayenne lay between these same clusters. Both cases suggest inclusion in a PCA cluster can be a coincidence of a line's relationships to other groups and PCA alone in these cases can be misleading.

Instances where specific information regarding pedigree or geographical origin was available, we found our analyses to be congruent with that information. The two Jalapeño and two Anaheim lines each form well supported clusters in all three analyses. The Bell line Jupiter was used as a recurrent backcross parent to generate Dempsey [58]. The phylogeny clusters these two lines with 90% support and the two lines were present in the Bell cluster by both PCA and Structure analysis. The *Phytophthora* resistant lines CM334 and PI201234 were each found to be genetically unique by these analyses. These lines are each derived from accessions collected in the states of Morelos in South-Central and Oaxaca in Southwestern Mexico, respectively [5]. Even though their fruit shape is similar and both lines are sources of *Phytophthora* resistance, the genetic basis of resistance differs between these two lines [59]. This along with their distinct geographical origins is consistent with the distinct genetic makeup identified in this analysis

The phylogeny shows a general clustering of lines across the panel but the relationships between the pungent lines was unresolved. This was not due solely to long branch attraction and/or the unresolved positions of CM334, PI201234 and Carolina Cayenne as the removal of these the lines from the analysis did not resolve the tree. The Structure analysis at K = 2 indicates that the ancestral population has a small contribution from the Bell cluster and large contribution from the Small hot cluster. This Small hot genomic contribution has been gradually lost during the selection for larger, fleshy, non-pungent fruits. This is in agreement with the overall trend during domestication and selection that has been previously observed [11], [22], [60]. This transition is clearly supported for the Anaheim/Ancho through Bell groups by Structure through K = 6, the phylogeny and the PCA with both the Structure and phylogenetic analyses indicating a grade of reduced diversity from the moderately fleshy, pungent Anaheim/Ancho types to the fleshy, non-pungent Bell types. Additionally, both the Structure and phylogenetic analyses indicate that the Bell types are derived from an ancestor in common with the Anaheim/Ancho types. This relationship was not apparent from previous studies [24]. The phylogenetic relationships among the remaining pungent types were unresolved and there was no clear ancestral type. This coincides with the Structure results at K = 6 where each of these pungent groups have distinct genomic contributions from different sets of clusters. A clear resolution of the relationships between these groups may require additional pungent types including semi-domesticated and wild *C. annuum*.

Only a few studies attempting to characterize a broad selection cultivated *C. annuum* genetic diversity using molecular markers have been reported. Each of these studies uses a small number (<150) of mostly anonymous markers [18]–[24]. The most comprehensive of these studies used retrotransposon LTRs to characterized 64 diverse *C. annuum* lines [24]. A Neighbor-Joining tree based on 107 polymorphic LTRs indicated four clusters among the *C. annuum* lines. Unlike our analyses, the pungent lines Jalapeño, CM334 and Perennial in the LTR-based analyses clustered together in the Neighbor-Joining tree and by Structure membership coefficients. This indicates that the high density of SPP markers provided higher resolution for analysis of population diversity and structure.

The growing affordability of high-throughput marker discovery has led to investigations into the most appropriate method for detecting population structure from large marker datasets [61]. Bayesian clustering and PCA are common methods and have been applied in several SNP studies of population structure in humans, livestock and crops [32], [62]–[64]. These methods having been developed for relatively small marker sets have limitations in their application using high density genome-wide markers [65]. The number of clusters estimated by Bayesian methods may be influenced by the inclusion of markers in linkage disequilibrium, an inevitability with large marker datasets [66]. PCA methods are sensitive to missing data and sampling effects for populations with continuous distributions [67]. Both methods can be affected by ascertainment bias, particularly when using SNP markers, due to unequal sampling across minor allele frequencies [68]. The SPP markers analyzed using Structure and PCA were detected *de novo* across the entire panel. This approach eliminates analytical issues associated with ascertainment bias. In addition, all markers with missing data were removed and, for the Structure analysis, the number of markers in linkage disequilibrium were reduced by removing SPPs with redundant allele profiles across the panel within each genetic bin. We found it critical to use the linkage model, applied to markers within mapped contigs. Without the benefit of map data, these analyses were more difficult to interpret (data not shown). An additional difficulty with Structure was in the estimation of K (the number of populations) since there was little difference in the posterior probabilities of each K [31], [52]. In this case it is suggested that the value of K that makes the most biological sense be used [31]. At each added K population, a high proportion of that population was assigned to an individual(s) that made biological sense up to K = 6, beyond which no large proportion was assigned any individual. Ultimately, this approach produced a high congruence of results between both the Structure and PCA analyses. The results of the Structure analysis appear most comprehensive, informative and in complete agreement with the phylogenetic analysis.

This work demonstrates that genome-wide SPP markers analyzed using three methods provided a clear description of diversity and relatedness among *Capsicum* breeding lines. A core collection of *Capsicum* has been established using clustering analysis of phenotypic characters [69]. A genome-wide marker analysis of this collection would extend our knowledge of the relatedness and diversity among its members. In addition to gene bank collections and modern cultivated lines, important sources of genetic diversity include the wild and semi-domesticated (land race) lines. A large number of wild and semi-domesticated land race populations of both *C. annuum* and *C. frutescens* are found growing in various environments from southern United States throughout Mexico [70]–[72]. It is clear that *C. annuum* was domesticated in Mexico, however the number and geographical centers of domestication events remain unclear. The application of the analyses implemented in study using a genome-wide set of SNP based markers to semi-domesticated and wild *annuums* may unravel the mysteries of *C. annuum* domestication. Understanding the populations derived from ancestors which have and have not contributed to modern breeding lines will aid in the selection of lines for future breeding programs. Additionally, identifying *C. annuum* populations that have contributed to modern domesticates may be required to more thoroughly dissect the genomes of and relationships between the cultivated pungent *annuums*.

Many of the traits of interest for current *Capsicum* breeding programs include complex, multi-genic traits that are not easily integrated through traditional breeding strategies. This is the first gene-based, genome-wide marker assessment of molecular diversity and population substructure among a broad collection of *C. annuum* lines. This work will complement our development of an ultra-high density pepper map and conversion to SNPs using the same chip technology enabling MAS for the introgression of complex traits, such as disease resistance, into the available breeding germplasm while retaining the integrity consumer driven traits.

## Supporting Information

Figure S1 **Pepper Chip assembly and probe representation.** (A) Distribution of contigs by size. (B) Distribution of probes per unigene on chip. The mean number of probes per unigene (210) is indicated with an arrow.
(TIF)

Figure S2 **Pepper GeneChip design and probe hybridization.** (A) The number of pepper genomic tiling probes by G/C content. (B) The number of anti-genomic background control probes per G/C content. (C) Percentage of probes above background per G/C content for 6 genotypes. (D) Hybridization difference between 2 genotypes at known SNPs by SNP position relative to probe center position. The weighting factor for probes is based on the equation shown above.
(TIF)

Figure S3 **Cluster analysis of replicate hybridizations across the pepper diversity panel.** A dendogram derived from 1000 bootstrap replicates of cluster analysis using the average method in the R package. The cluster analysis was carried out using 5431 probes identified as polymorphic across the diversity panel. Green numbers represent the percent bootstrap support. The three replicate chips for each genotype (GT#) clustered together with high bootstrap support across all genotypes with the exception of the closely related Bell types Bruinsma Wonder (GT03015) and Charleston Belle (GT03018) which were not separated by this analysis.
(TIF)

Figures S4 **Data flow diagram.** A flow diagram showing the major steps in data acquisition, SPP detection and data filtering prior to analyses.
(PDF)

Figure S5 **Effect of filtering on SPP detection and false positive rate.** (A) The number of SPPs and unigenes identified at a minimum SPPdev ratio of 1.5 while varying minimum number of probes and bases required for detection with zero inconsistent (I) and missing (-) calls allowed. (B) The number of SPPs and unigenes identified at increasing minimum SPPdev ratios with minimum requirements of 4 bases spanned, 2 informative probes and zero I and - allowed. (C) Allowing inconsistent (I) calls where% Inconsistent is the maximum percentage of I calls allowed across all 43 lines per SPP marker. (D) False positive rates at minimums of 2 and 4 bases spanned by an SPP at minimum SPPdev ratio of 1.5 while varying missing (M) and inconsistent (I) calls per SPP allowed. (E) False positive rates with and without SPPs identified in duplicated sequences included at 2 probes and 4 bases spanned while varying SPPdev ratio minimums and I calls allowed.
(TIF)

Figure S6 **Genotyping at the** ***Pun1*** **locus.** PCR using primers spanning the *pun1^1^* deletion were used to determine *Pun1* genotype for all lines. Varieties with the ∼250 bp band carry the non-functional *pun1^1^* deletion.
(TIF)

Figure S7 **Cluster analysis using the Ward method of the first 3 principle components.** Ward clustering of Prin 1, Prin 2, and Prin 3 eigenvalues identified by Principle Component Analysis (PCA) of 3818 unigenes (6,426 SPPs) across 40 *C. annuum* lines.
(TIF)

Table S1 **Pairwise T-tests on data filtering.** Levels not connected by same letter are significantly different at 1p<0.01, 2p<0.0001.
(PPT)

Table S2 **Accuracy of SPP calls.** Using allele-specific PCR, comparison of 27 SPPs/SNPs x 43 genotypes for a total of 1161 allele calls. SPP dataset: Min SFPdev Ratio = 1.2, 2 probes, 4 bases.
(PPT)

Table S3 **Pairwise T-tests on principle components, genotypes grouped by Structure clusters.** Levels not connected by same letter are significantly different. p<0.01.
(PPT)

Table S4 **Hybridization efficiency of Solunum spp.** Probes > background  =  probes hybridized at levels greater than the 90th percentile of anti-genomic probes and Unigenes represented  =  the number of unigenes with probes hybridized above background levels.
(PPT)

Dataset S1 **Unigenes used to design the Pepper GeneChip.** Each unigene was designated a unique SequenceID. The length of each unigene is given. Probe/SPP positions align with the unigenes with the first nt of each unigene designated position 1.
(XLSB)

Dataset S2 **Polymorphic SPPs identified at SPPdev 1.2 used for diversity analysis.** Allele calls for each of 33,401 SPPs are shown. Allele frequencies across the panel at each SPP are also given. The dataset was generated using the following detection/filter settings: SPPdev 1.2, minimum 2 informative probes, minimum 4 bases spanned, Ds and Cs were converted to As and Bs, respectively, a minimum each of A or B of 1, 0 missing, 0 I, SPPs within duplicate regions were removed.
(XLSB)

Dataset S3 **Polymorphic SPPs identified across the 43 pepper varieties at SPPdev 1.2.** Allele calls for each line at each of 103,863 SPPs are shown. Allele frequencies across the panel at each SPP are also given. The dataset was generated using the following detection/filter settings: SPPdev 1.2, minimum 2 informative probes, minimum 4 bases spanned, minimum each A+D (“A”) and B+C (“B”) of 1, maximum missing 96%, maximum I 40%.
(XLSB)

Dataset S4 **Polymorphic SPPs identified across the 43 pepper varieties at SPPdev 1.5.** Allele calls for each of 140,548 SPPs are shown. Allele frequencies across the panel at each SPP are also given. The dataset was generated using the following detection/filter settings: SPPdev 1.5, minimum 2 informative probes, minimum 2 bases spanned, minimum each A+D (“A”) and B+C (“B”) of 1, maximum missing 96%, maximum I 40%.
(XLSB)

Dataset S5 **Polymorphic SPPs identified across the 43 pepper varieties at SPPdev 2.0.** Allele calls for each of 24,592 SPPs are shown. The dataset was generated using the following detection/filter settings: SPPdev 2.0, minimum 2 informative probes, minimum 4 bases spanned, minimum each A+D (“A”) and B+C (“B”) of 1, maximum missing 96%, maximum I 40%.
(XLSB)

Dataset S6 **1729 Informative SPPs across pungent and non-pungent** ***C. annuum*** **lines.** Allele calls for the subset of SPP markers in Dataset S2 that are polymorphic within both pungent and non-pungent groups. A and B allele frequencies and minor allele frequencies across the panel and within pungent and non-pungent *C. annuum* and non- annuum C. spp. at each SPP are also given.
(XLSB)
