# Supplementary figures and images for: Characterization of Capsicum annuum Genetic Diversity and Population Structure Based on Parallel Polymorphism Discovery with a 30K Unigene Pepper GeneChip
Source: PLoS One. 2013 Feb 8;8(2):e56200. doi: 10.1371/journal.pone.0056200 (PMC3568043; doi:10.1371/journal.pone.0056200)

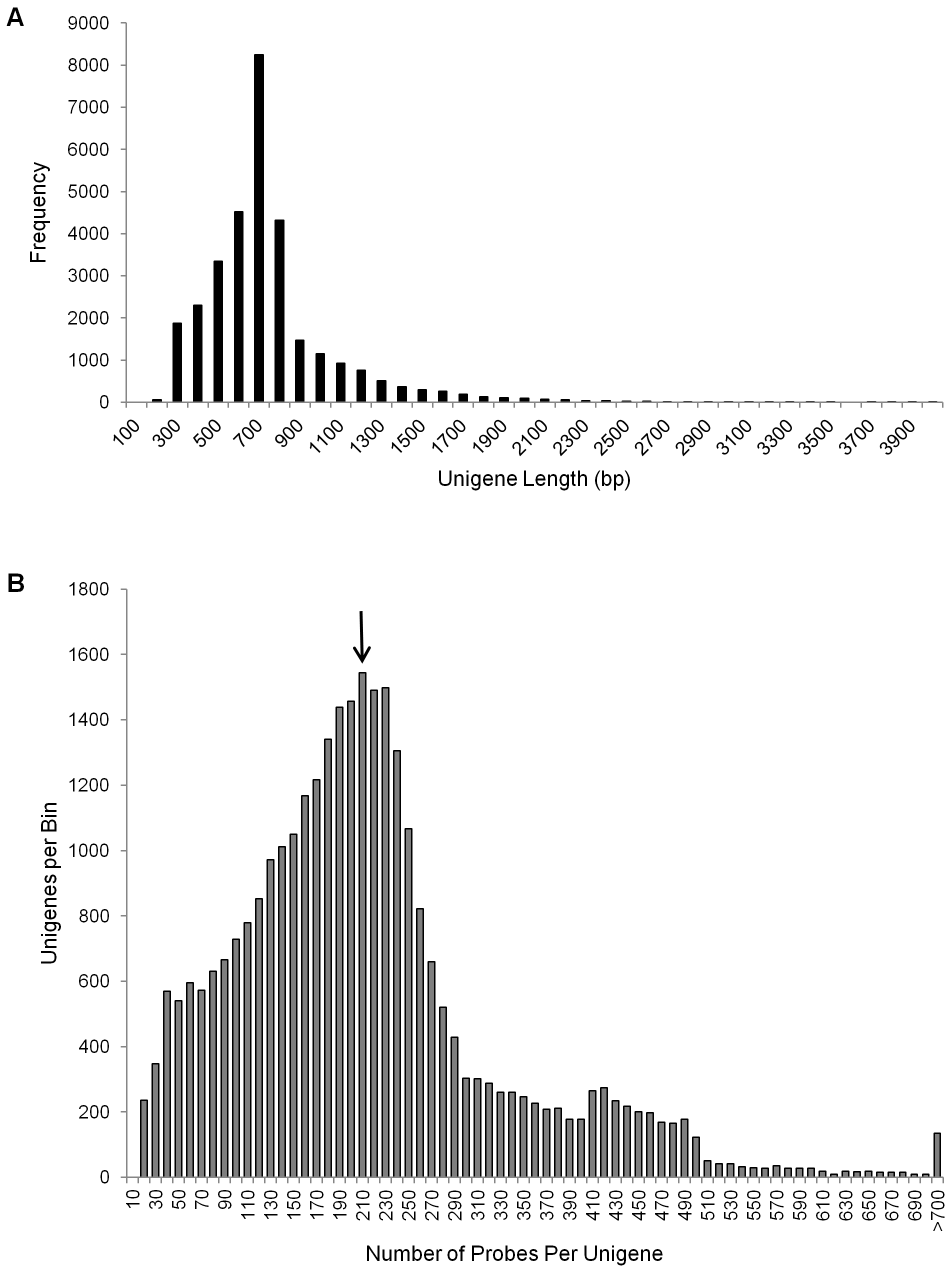

Supplement: Figure S1 — Pepper Chip assembly and probe representation. (A) Distribution of contigs by size. (B) Distribution of probes per unigene on chip. The mean number of probes per unigene (210) is indicated with an arrow. (TIF) [file pone.0056200.s001.tif]

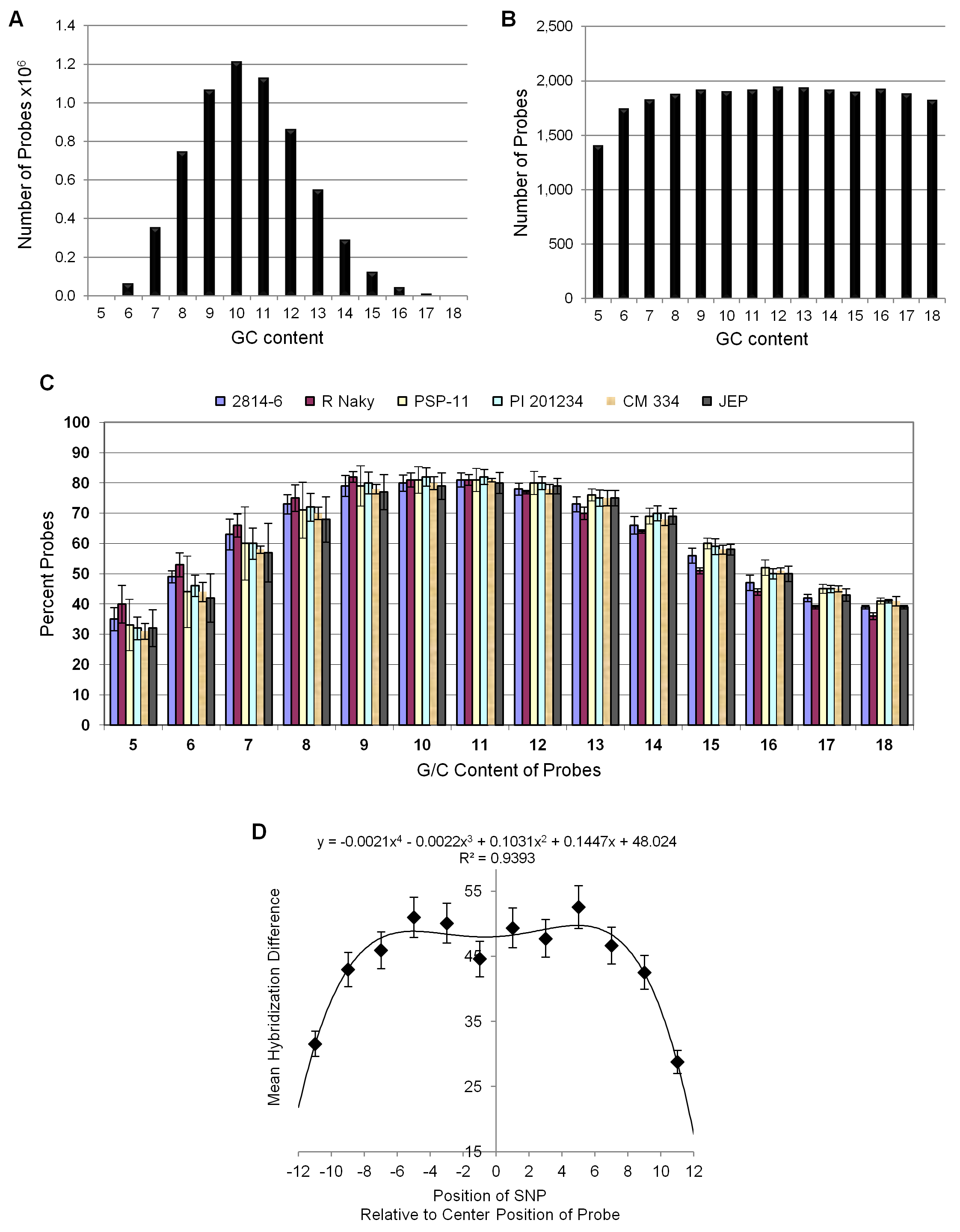

Supplement: Figure S2 — Pepper GeneChip design and probe hybridization. (A) The number of pepper genomic tiling probes by G/C content. (B) The number of anti-genomic background control probes per G/C content. (C) Percentage of probes above background per G/C content for 6 genotypes. (D) Hybridization difference between 2 genotypes at known SNPs by SNP position relative to probe center position. The weighting factor for probes is based on the equation shown above. (TIF) [file pone.0056200.s002.tif]

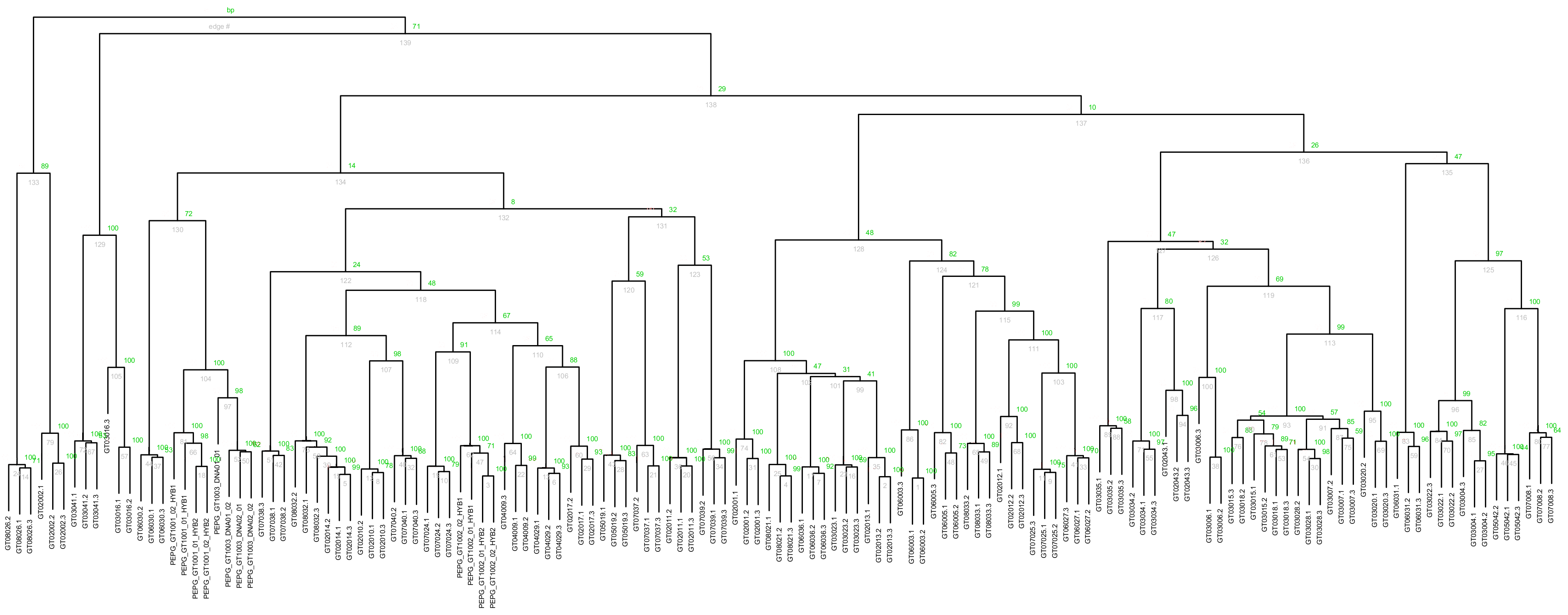

Supplement: Figure S3 — Cluster analysis of replicate hybridizations across the pepper diversity panel. A dendogram derived from 1000 bootstrap replicates of cluster analysis using the average method in the R package. The cluster analysis was carried out using 5431 probes identified as polymorphic across the diversity panel. Green numbers represent the percent bootstrap support. The three replicate chips for each genotype (GT#) clustered together with high bootstrap support across all genotypes with the exception of the closely related Bell types Bruinsma Wonder (GT03015) and Charleston Belle (GT03018) which were not separated by this analysis. (TIF) [file pone.0056200.s003.tif]

Data acquisition

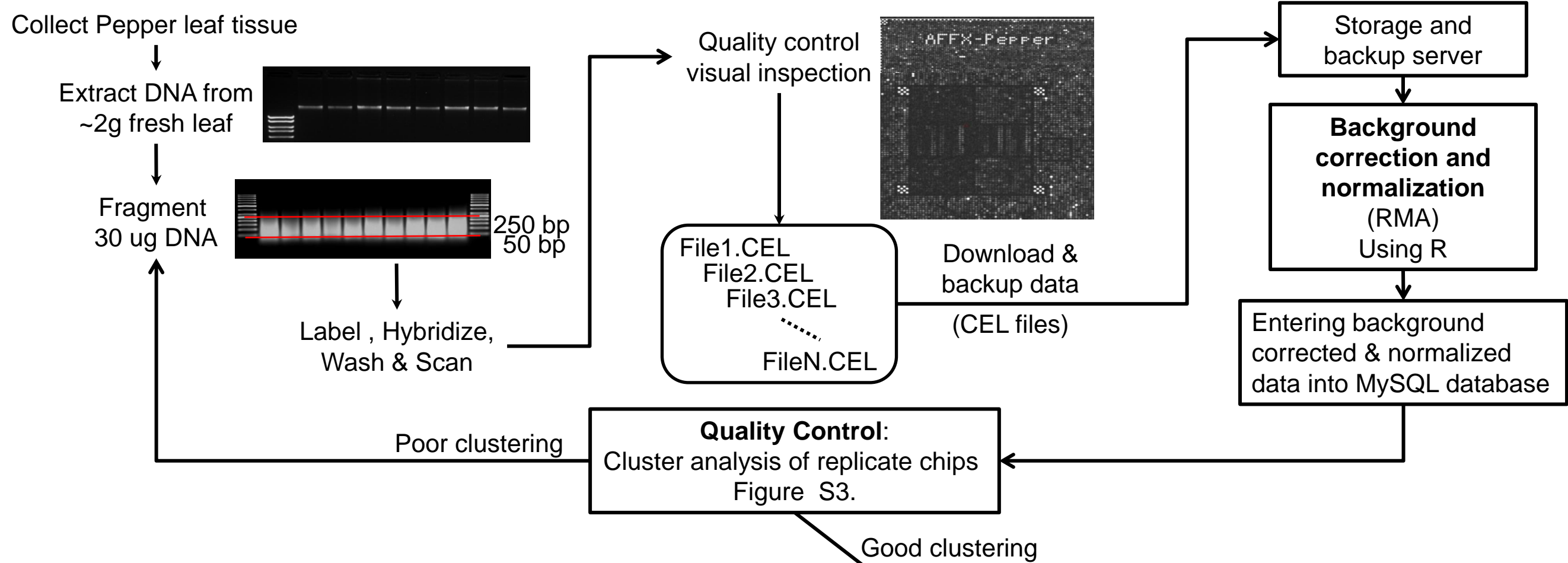

Data analysis

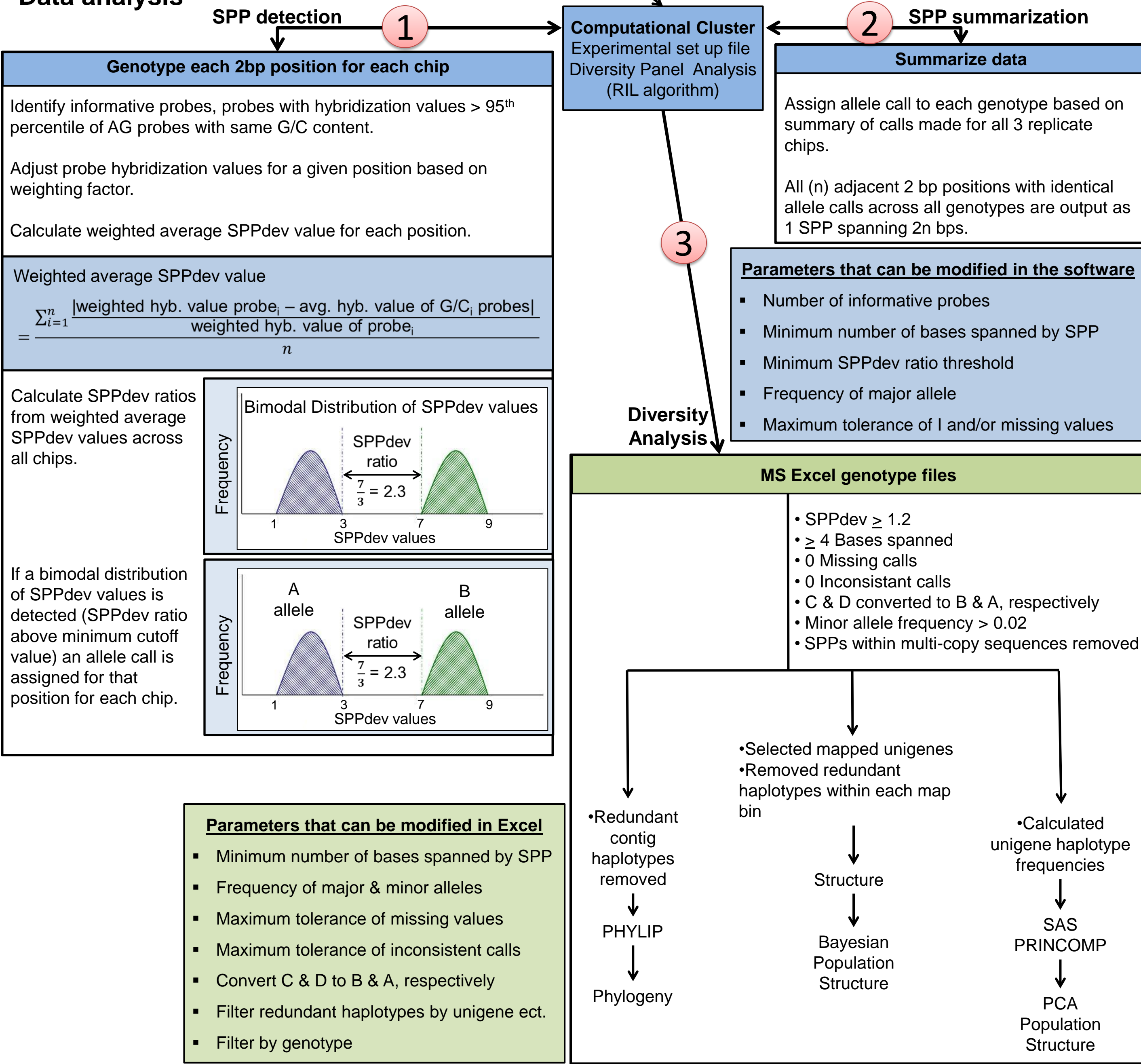

Supplement: Figures S4 — Data flow diagram. A flow diagram showing the major steps in data acquisition, SPP detection and data filtering prior to analyses. (PDF) [file pone.0056200.s004.pdf]

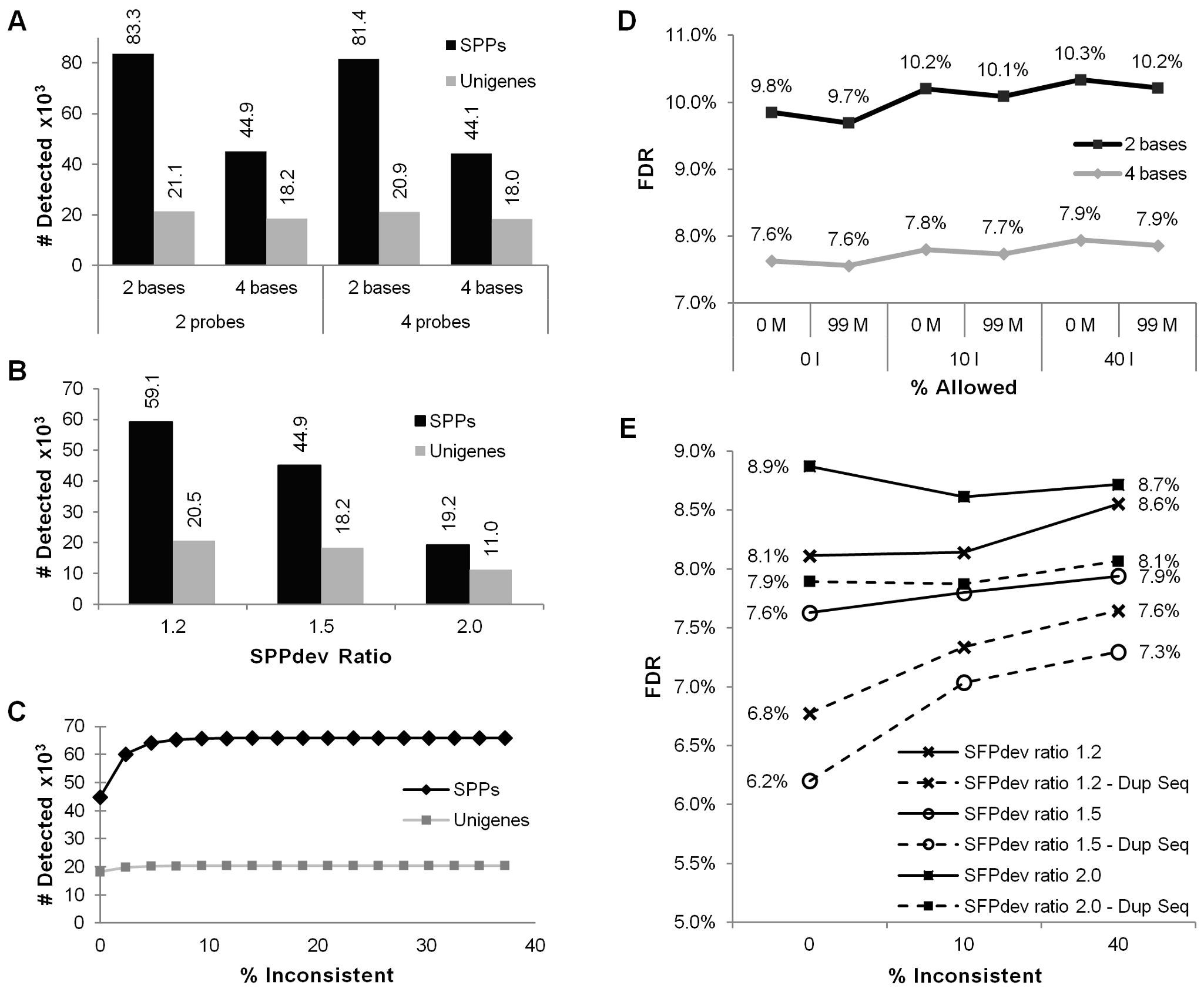

Supplement: Figure S5 — Effect of filtering on SPP detection and false positive rate. (A) The number of SPPs and unigenes identified at a minimum SPPdev ratio of 1.5 while varying minimum number of probes and bases required for detection with zero inconsistent (I) and missing (-) calls allowed. (B) The number of SPPs and unigenes identified at increasing minimum SPPdev ratios with minimum requirements of 4 bases spanned, 2 informative probes and zero I and - allowed. (C) Allowing inconsistent (I) calls where% Inconsistent is the maximum percentage of I calls allowed across all 43 lines per SPP marker. (D) False positive rates at minimums of 2 and 4 bases spanned by an SPP at minimum SPPdev ratio of 1.5 while varying missing (M) and inconsistent (I) calls per SPP allowed. (E) False positive rates with and without SPPs identified in duplicated sequences included at 2 probes and 4 bases spanned while varying SPPdev ratio minimums and I calls allowed. (TIF) [file pone.0056200.s005.tif]

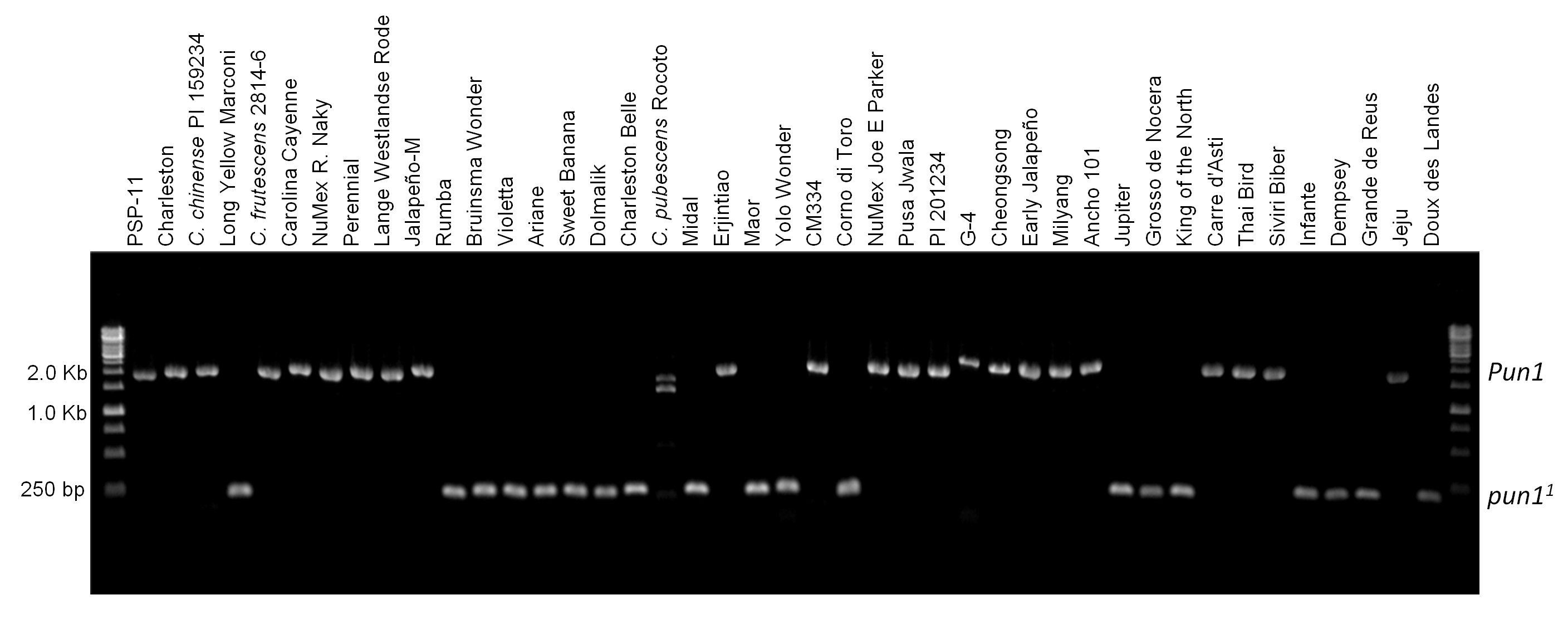

Supplement: Figure S6 — Genotyping at the Pun1 locus. PCR using primers spanning the pun11 deletion were used to determine Pun1 genotype for all lines. Varieties with the ∼250 bp band carry the non-functional pun11 deletion. (TIF) [file pone.0056200.s006.tif]

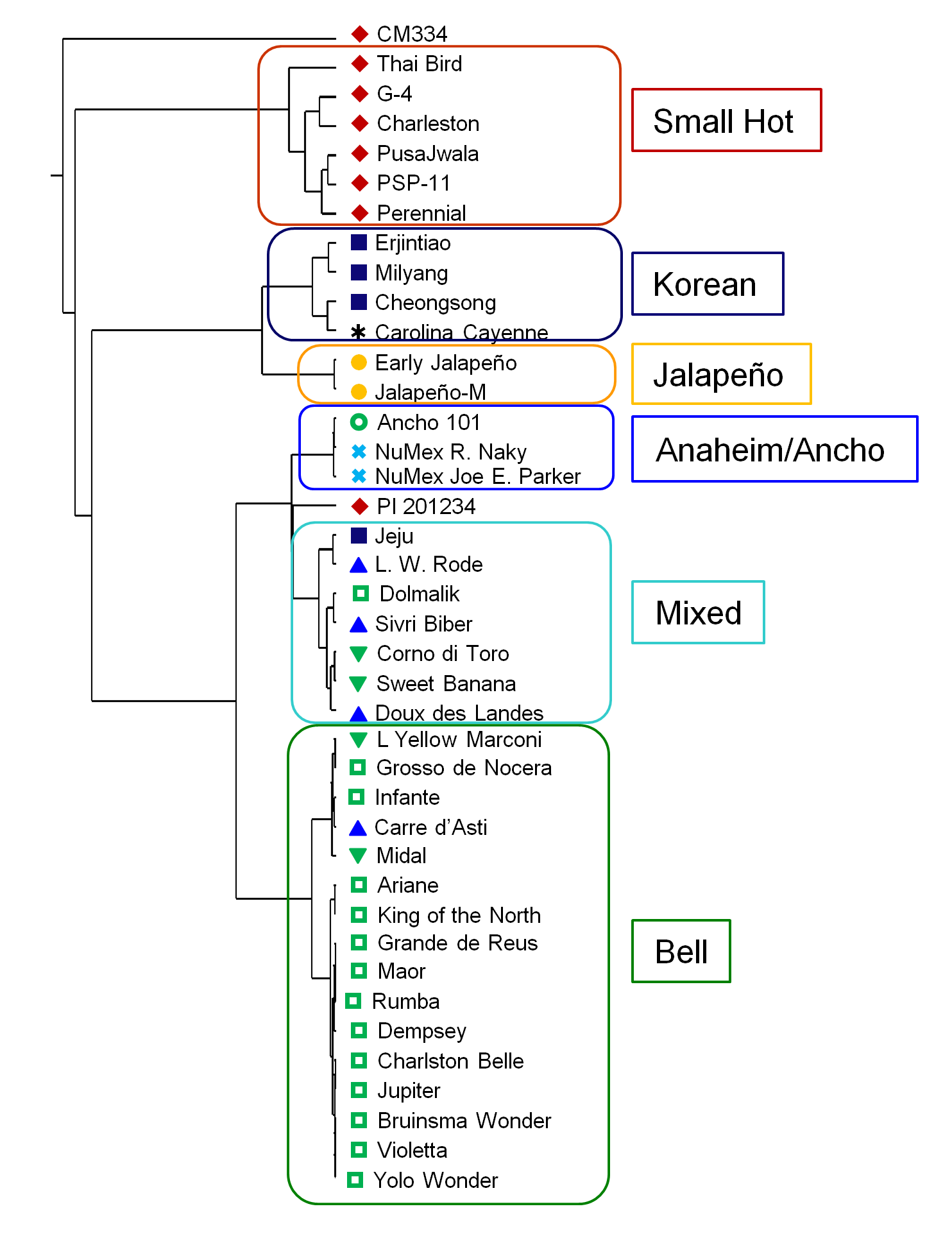

Supplement: Figure S7 — Cluster analysis using the Ward method of the first 3 principle components. Ward clustering of Prin 1, Prin 2, and Prin 3 eigenvalues identified by Principle Component Analysis (PCA) of 3818 unigenes (6,426 SPPs) across 40 C. annuum lines. (TIF) [file pone.0056200.s007.tif]
