# Supplementary material for: Characterization of Capsicum annuum Genetic Diversity and Population Structure Based on Parallel Polymorphism Discovery with a 30K Unigene Pepper GeneChip
Source: PLoS One. 2013 Feb 8;8(2):e56200. doi: 10.1371/journal.pone.0056200 (PMC3568043; doi:10.1371/journal.pone.0056200)
Supplement: Table S1 — Pairwise T-tests on data filtering. Levels not connected by same letter are significantly different at 1p<0.01, 2p<0.0001. (PPT) [file pone.0056200.s008.ppt]

## Slide 1
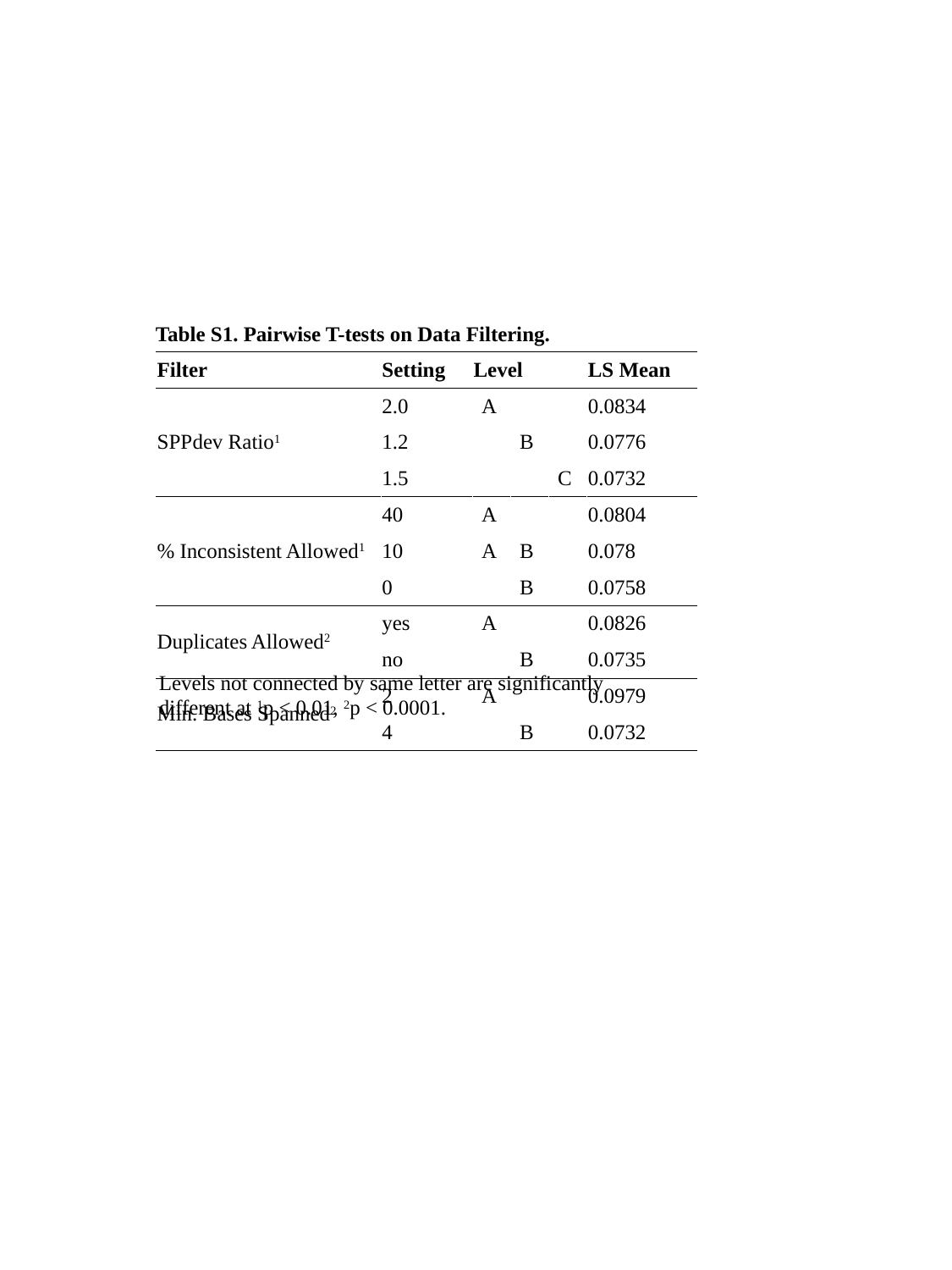

Table S1. Pairwise T-tests on Data Filtering.
| Filter | Setting | Level | | | LS Mean |
| --- | --- | --- | --- | --- | --- |
| SPPdev Ratio1 | 2.0 | A | | | 0.0834 |
| | 1.2 | | B | | 0.0776 |
| | 1.5 | | | C | 0.0732 |
| % Inconsistent Allowed1 | 40 | A | | | 0.0804 |
| | 10 | A | B | | 0.078 |
| | 0 | | B | | 0.0758 |
| Duplicates Allowed2 | yes | A | | | 0.0826 |
| | no | | B | | 0.0735 |
| Min. Bases Spanned2 | 2 | A | | | 0.0979 |
| | 4 | | B | | 0.0732 |
Levels not connected by same letter are significantly different at 1p < 0.01, 2p < 0.0001.
