# Supplementary material for: Characterization of Capsicum annuum Genetic Diversity and Population Structure Based on Parallel Polymorphism Discovery with a 30K Unigene Pepper GeneChip
Source: PLoS One. 2013 Feb 8;8(2):e56200. doi: 10.1371/journal.pone.0056200 (PMC3568043; doi:10.1371/journal.pone.0056200)
Supplement: Table S2 — Accuracy of SPP calls. Using allele-specific PCR, comparison of 27 SPPs/SNPs x 43 genotypes for a total of 1161 allele calls. SPP dataset: Min SFPdev Ratio = 1.2, 2 probes, 4 bases. (PPT) [file pone.0056200.s009.ppt]

## Slide 1
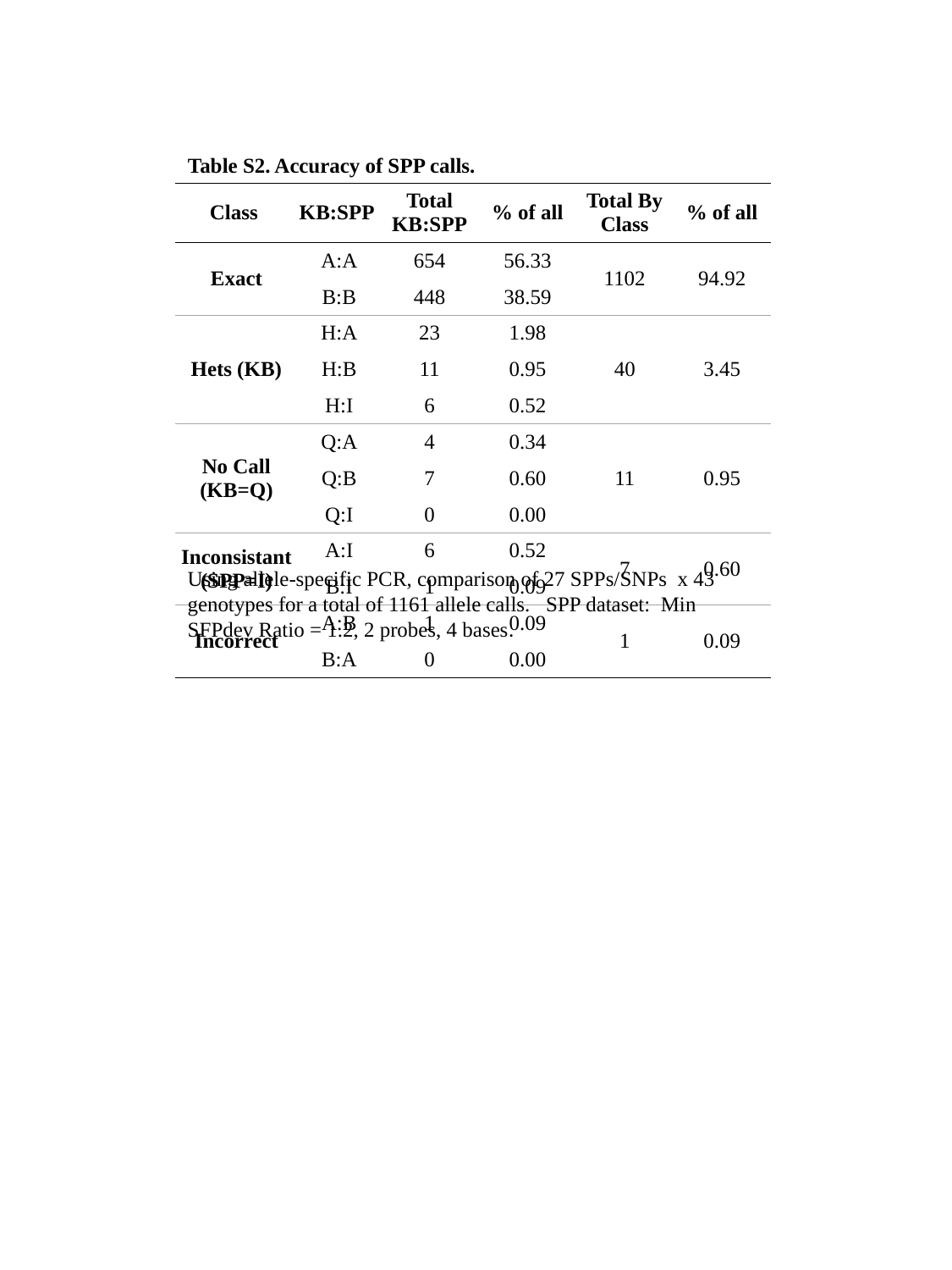

Table S2. Accuracy of SPP calls.
| Class | KB:SPP | Total KB:SPP | % of all | Total By Class | % of all |
| --- | --- | --- | --- | --- | --- |
| Exact | A:A | 654 | 56.33 | 1102 | 94.92 |
| | B:B | 448 | 38.59 | | |
| Hets (KB) | H:A | 23 | 1.98 | 40 | 3.45 |
| | H:B | 11 | 0.95 | | |
| | H:I | 6 | 0.52 | | |
| No Call (KB=Q) | Q:A | 4 | 0.34 | 11 | 0.95 |
| | Q:B | 7 | 0.60 | | |
| | Q:I | 0 | 0.00 | | |
| Inconsistant (SPP=I) | A:I | 6 | 0.52 | 7 | 0.60 |
| | B:I | 1 | 0.09 | | |
| Incorrect | A:B | 1 | 0.09 | 1 | 0.09 |
| | B:A | 0 | 0.00 | | |
Using allele-specific PCR, comparison of 27 SPPs/SNPs x 43 genotypes for a total of 1161 allele calls. SPP dataset: Min SFPdev Ratio = 1.2, 2 probes, 4 bases.
