# Supplementary material for: Characterization of Capsicum annuum Genetic Diversity and Population Structure Based on Parallel Polymorphism Discovery with a 30K Unigene Pepper GeneChip
Source: PLoS One. 2013 Feb 8;8(2):e56200. doi: 10.1371/journal.pone.0056200 (PMC3568043; doi:10.1371/journal.pone.0056200)
Supplement: Table S3 — Pairwise T-tests on principle components, genotypes grouped by Structure clusters. Levels not connected by same letter are significantly different. p<0.01. (PPT) [file pone.0056200.s010.ppt]

## Slide 1
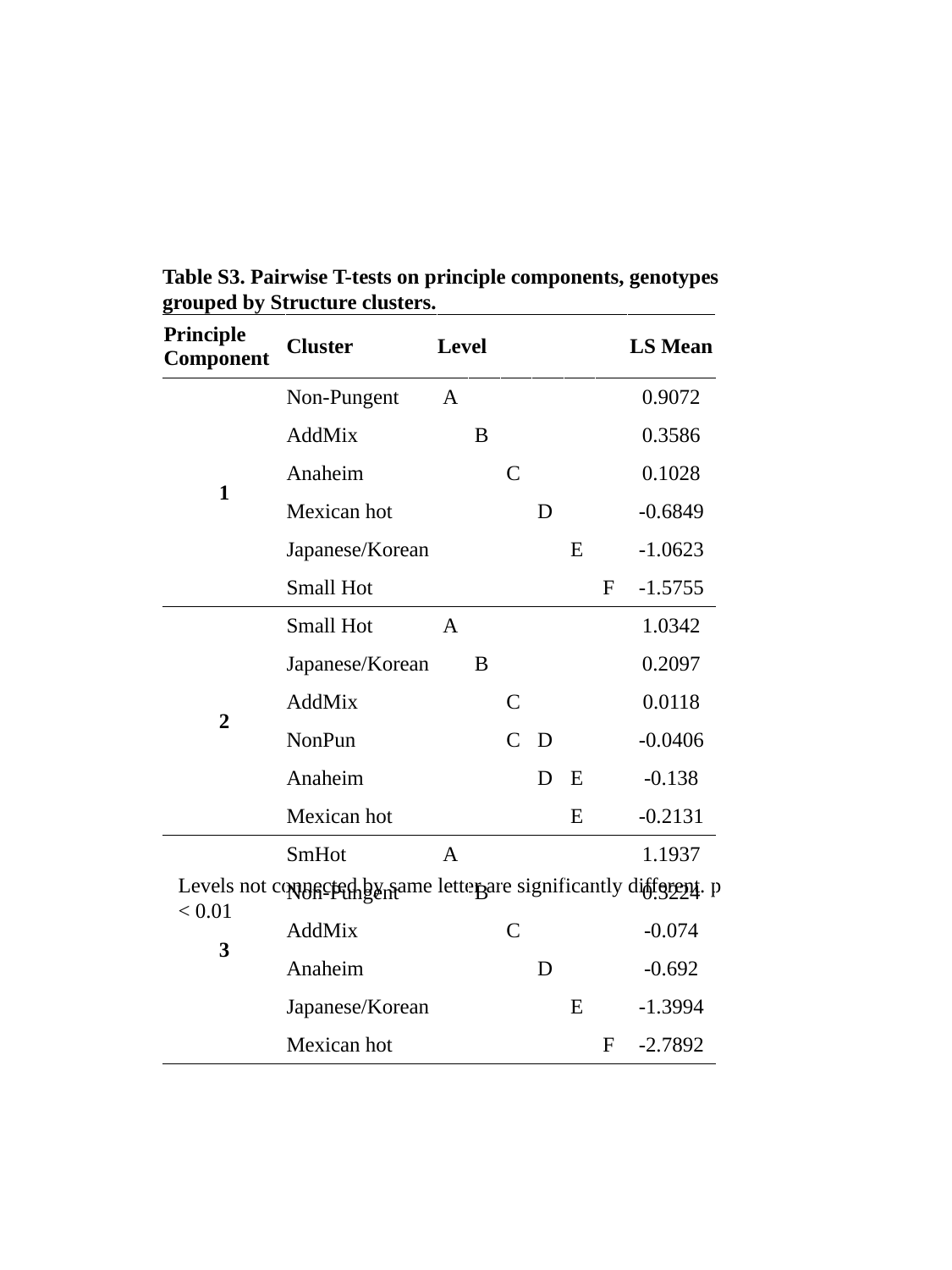

Table S3. Pairwise T-tests on principle components, genotypes grouped by Structure clusters.
| Principle Component | Cluster | Level | | | | | | LS Mean |
| --- | --- | --- | --- | --- | --- | --- | --- | --- |
| 1 | Non-Pungent | A | | | | | | 0.9072 |
| | AddMix | | B | | | | | 0.3586 |
| | Anaheim | | | C | | | | 0.1028 |
| | Mexican hot | | | | D | | | -0.6849 |
| | Japanese/Korean | | | | | E | | -1.0623 |
| | Small Hot | | | | | | F | -1.5755 |
| 2 | Small Hot | A | | | | | | 1.0342 |
| | Japanese/Korean | | B | | | | | 0.2097 |
| | AddMix | | | C | | | | 0.0118 |
| | NonPun | | | C | D | | | -0.0406 |
| | Anaheim | | | | D | E | | -0.138 |
| | Mexican hot | | | | | E | | -0.2131 |
| 3 | SmHot | A | | | | | | 1.1937 |
| | Non-Pungent | | B | | | | | 0.3224 |
| | AddMix | | | C | | | | -0.074 |
| | Anaheim | | | | D | | | -0.692 |
| | Japanese/Korean | | | | | E | | -1.3994 |
| | Mexican hot | | | | | | F | -2.7892 |
Levels not connected by same letter are significantly different. p < 0.01
