# Supplementary material for: Characterization of Capsicum annuum Genetic Diversity and Population Structure Based on Parallel Polymorphism Discovery with a 30K Unigene Pepper GeneChip
Source: PLoS One. 2013 Feb 8;8(2):e56200. doi: 10.1371/journal.pone.0056200 (PMC3568043; doi:10.1371/journal.pone.0056200)
Supplement: Table S4 — Hybridization efficiency of Solunum spp. Probes > background = probes hybridized at levels greater than the 90th percentile of anti-genomic probes and Unigenes represented = the number of unigenes with probes hybridized above background levels. (PPT) [file pone.0056200.s011.ppt]

## Slide 1
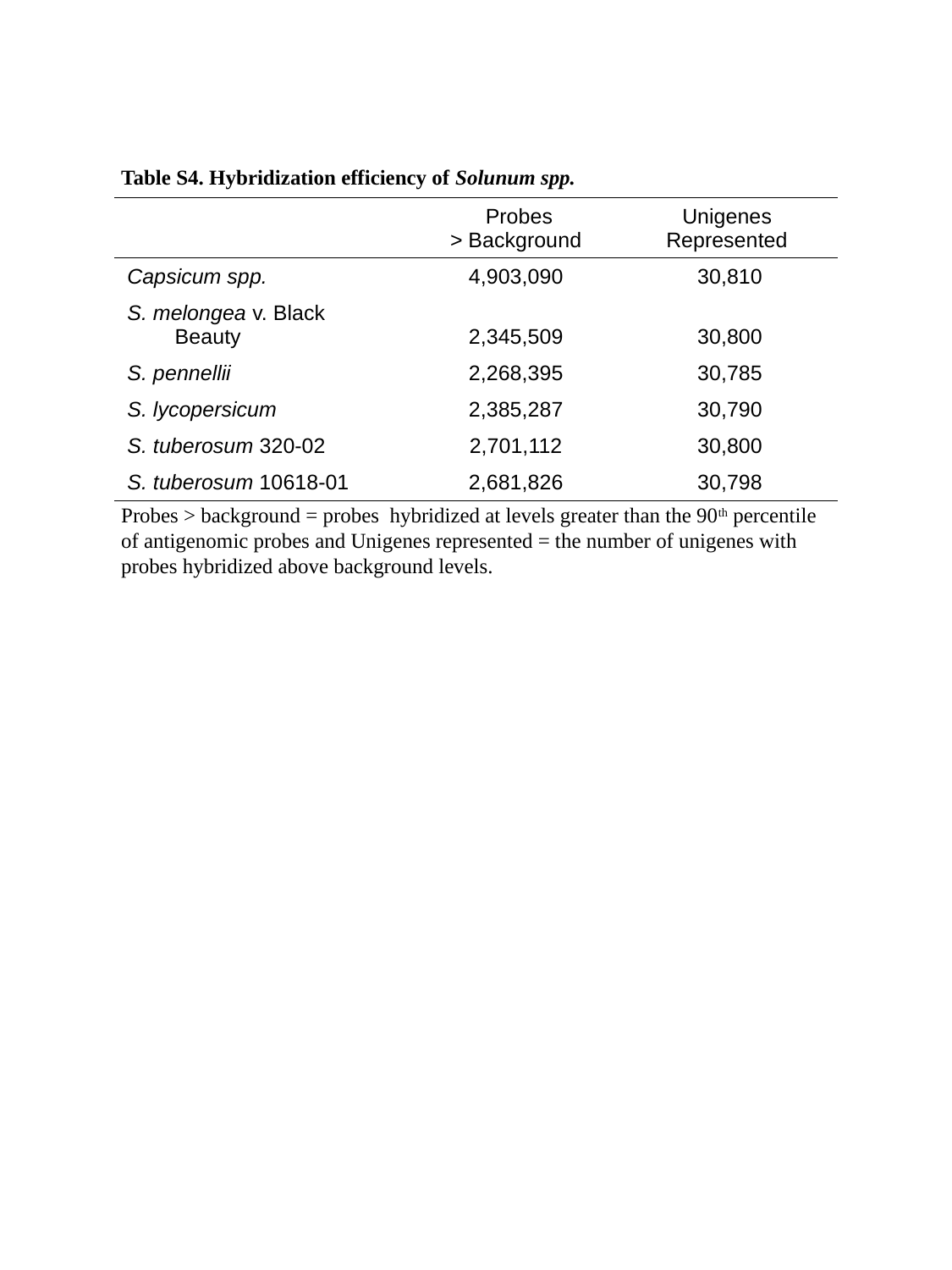

Table S4. Hybridization efficiency of Solunum spp.
| | Probes > Background | Unigenes Represented |
| --- | --- | --- |
| Capsicum spp. | 4,903,090 | 30,810 |
| S. melongea v. Black Beauty | 2,345,509 | 30,800 |
| S. pennellii | 2,268,395 | 30,785 |
| S. lycopersicum | 2,385,287 | 30,790 |
| S. tuberosum 320-02 | 2,701,112 | 30,800 |
| S. tuberosum 10618-01 | 2,681,826 | 30,798 |
Probes > background = probes hybridized at levels greater than the 90th percentile of antigenomic probes and Unigenes represented = the number of unigenes with probes hybridized above background levels.
